# Large-scale evaluation of dynamically important residues in proteins predicted by the perturbation analysis of a coarse-grained elastic model

**DOI:** 10.1186/1472-6807-9-45

**Published:** 2009-07-10

**Authors:** Wenjun Zheng, Mustafa Tekpinar

**Affiliations:** 1 Physics Department, University at Buffalo, Buffalo, NY 14260, USA

## Abstract

**Backgrounds:**

It is increasingly recognized that protein functions often require intricate conformational dynamics, which involves a network of key amino acid residues that couple spatially separated functional sites. Tremendous efforts have been made to identify these key residues by experimental and computational means.

**Results:**

We have performed a large-scale evaluation of the predictions of dynamically important residues by a variety of computational protocols including three based on the perturbation and correlation analysis of a coarse-grained elastic model. This study is performed for two lists of test cases with >500 pairs of protein structures. The dynamically important residues predicted by the perturbation and correlation analysis are found to be strongly or moderately conserved in >67% of test cases. They form a sparse network of residues which are clustered both in 3D space and along protein sequence. Their overall conservation is attributed to their dynamic role rather than ligand binding or high network connectivity.

**Conclusion:**

By modeling how the protein structural fluctuations respond to residue-position-specific perturbations, our highly efficient perturbation and correlation analysis can be used to dissect the functional conformational changes in various proteins with a residue level of detail. The predictions of dynamically important residues serve as promising targets for mutational and functional studies.

## Background

Protein conformational dynamics [1,2] is critically involved in many biochemical processes ranging from catalysis [3] to allostery [4-9] and signal transduction [10]. Protein dynamics spans a wide range of time scales (from picoseconds to seconds or minutes). Biologically relevant conformational motions of proteins are often collective (for example in the form of hinge-bending or shearing motions between rigid domains, see [11]). These highly coordinated motions are thought to involve a network of key amino acid residues that couple spatially separated functional sites [9]. The conservation and variation of protein functions are likely underscored by the conservation and co-evolution of these dynamically important residues. The existence of a sparse network of allosterically coupled residues in various proteins has been revealed by the statistical coupling (or correlated mutation) analysis based on multiple sequence alignment (see [12-14]). The discovery of dynamically important residues will lead to the following important applications: first, facilitate the mechanistic studies of a variety of biomolecular systems whose conformational dynamics plays a key role in function [1]; second, enable drug design that dynamically alters target proteins via small molecule binding [15-18]; third, guide the engineering of new molecular devices with novel dynamic properties [19,20].

In complement with experimental efforts for probing protein dynamics at atomic resolution (such as NMR, see [21,22]; and time-resolved Xray crystallography, see [23]), structure-based computer simulations have promised to elucidate the fine details of protein conformational motions. When multiple crystal structures are available for a protein at different states, structural analysis can identify those residues involved in the functional conformational changes between these states (such as local motions in allosteric proteins, see [24]; conformational changes due to binding of small molecules and other proteins, see [25,26]). In one study, the analysis of local structural changes (such as changes in the pseudo-bond angles and pseudo-dihedral angles along the C_*α *_trace of a protein) was employed to identify key residues involved in the lid-closing conformational change in adenylate kinase [27]. Hinge residues of protein domain motions can be identified by structural analysis tools such as DynDom [28] and Hingefind [29], or graph theory based method [30]. To further yield dynamic information from static crystal structures, atomistic molecular dynamics (MD) [31] and related methods (such as non-equilibrium MD, see [32]; targeted MD, see [33]; biased MD, see [34]) have been employed to identify key residues or structural elements involved in protein conformational fluctuations and transitions (see [32,35,36]). Nevertheless, the applications of atomistic MD simulations are limited by the high cost of simulating conformational dynamics beyond tens of nanoseconds [31].

To overcome the time-scale barrier for atomistic MD simulations, a variety of coarse-grained (low-resolution) modeling techniques [37] have been developed to explore protein conformational motions more efficiently. For example, the Go model [38] has been recently used to simulate conformational transitions between known protein structures (see [39]). A structural thermodynamic model (COREX) based on "Go-like" sampling of protein ensembles was developed to simulate coupling between local structural fluctuations, ligand binding and global conformational changes [40]. Of particular interest to the present study is the elastic network model (ENM) [41-43] and its isotropic variation – Gaussian network model (GNM) [44,45], which represent a protein structure as a network of C_*α *_atoms locally connected by springs with a uniform force constant [46]. The normal mode analysis (NMA) of ENM often yields a handful of low-frequency modes that dominate the large-scale domain motions observed between protein crystal structures [43,47,48]. Numerous studies have established ENM as an efficient means to tease out the functionally relevant conformational dynamics from protein structures with no limit in time scale or system size (for reviews, see [49-51]). The ENM/GNM-based NMA has formed the basis of several recently developed computational methods for locating ligand-binding sites [52,53], predicting hinge residues using low-frequency modes [54-56], and modeling conformational transition pathways [57-61]. Thanks to its high efficiency the ENM/GNM-based NMA has proven to be a powerful tool for bioinformatic analysis of protein structures and motions at a large scale [47,48,62].

In several recent studies, we have proposed and employed an ENM-based perturbation analysis to predict the dynamically important residues involved in the observed protein functional motions [63-65]. This method analyzes how the "dominant mode" (the normal mode that dominates the observed protein functional motions) changes in response to residue-position-specific perturbations to the ENM force constant that mimic the effects of point mutations (see Methods). By combining the perturbation analysis with the linear response theory, a correlation analysis was developed to locate hinge residues that control the structural fluctuations of the entire protein structure or its active site [66,67]. These novel methods are available at an NIH-based webserver . In our previous studies of chaperonin GroEL, helicase, myosin and polymerases [63-67], we have shown that the key residues involved in the functional motions of these proteins are highly conserved, and many of them were found to be functionally important by mutational studies [63-65]. Nevertheless, the applicability of these methods and other coarse-grained methods to a large variety of proteins remains to be established. Additionally, the perturbation analysis was based on a single dominant mode, while the conformational changes in proteins often involve multiple modes [47,48]. So it is necessary to extend our method to study protein functional motions that are not dominated by a single normal mode.

In this study, we will first introduce a new fluctuation-based perturbation protocol (see Methods) that does not assume the existence of a single dominant mode. Then we will perform a comprehensive evaluation of our ENM-based methods together with several alternative ones for predicting dynamically important residues. For the lack of systematic data on protein dynamics and functions, we will use sequence conservation as the primary metric for method evaluation. The evaluation is performed on two lists of protein structure pairs – a short list with 25 pairs of protein structures which is compiled from previous works by us and others [43,68,69]; a long list with >500 pairs gleaned from Protein Data Bank by an automated procedure (see Methods). The above two lists are complementary in the following ways. The short list contains functionally relevant structural changes whose biological significance was established in literature (all of them are involved in the binding/release of a biologically relevant ligand), but the arbitrary selection of a small number of cases may introduce artificial bias and statistical uncertainty; the long list, however, may contain biologically irrelevant structural changes (for example, due to crystal packing), but it provides a less biased data set with smaller statistical errors.

We have found that the dynamically important residues predicted by the perturbation and correlation analysis are strongly or moderately conserved for >67% of the test cases. These key residues, which constitute a small fraction of all residues (~15%), are clustered both in 3D space and along protein sequence, and they dominate the structural dynamics of ENM. Together, they form a sparse network which may couple distant functional sites in a protein complex. The conservation of these key residues is mainly due to their dynamic role instead of ligand binding or high network connectivity. This large-scale study, along with previous database-scale studies using NMA [47,62,70-72], will pave the way for future bioinformatic analyses of protein structure-function relationships via high-throughput dynamic modeling. These techniques promise to offer new biophysical insights that will enrich the existing databases of protein structures and motions [71].

## Methods

### 1. Elastic Network Model (ENM)

In an ENM, a protein structure is represented as a network of C_*α *_atoms of amino acid residues whose equilibrium coordinates are given by a crystal structure. A harmonic potential with a uniform force constant *C *accounts for elastic interactions between two C_*α *_atoms that lie within a cutoff distance *R*_*c *_(it is usually set within the range of 7 Å–20 Å). The potential energy function of ENM is [46]

(1)

where *d*_*ij *_is the distance between the C_*α *_atom *i *and *j*,  is the equilibrium distance between the C_*α *_atom *i *and *j *in the crystal structure, *N *is the number of C_*α *_atoms, and *θ *(*x*) is the Heaviside function.

The above potential energy function is expanded to second order:

(2)

where *δX *= *X *- *X*_0_, *X *is a 3*N*-dimensional vector representing the Cartesian coordinates of *N *C_*α *_atoms, C_*α *_gives the equilibrium C_*α *_coordinates in the crystal structure,  is the Hessian matrix, and the matrix element of *H*^*ij *^is given by , where *a *and *b *are indices for x, y and z components of the Cartesian coordinates of C_*α *_atoms *i*' and *j*'.

A normal mode analysis of the Hessian matrix yields 3*N*-6 non-zero normal modes (excluding 6 zero modes corresponding to 3 rotations and 3 translations), which are numbered from 1 to 3*N*-6 in order of ascending eigenvalue. To validate ENM, each normal mode is compared with the observed structural change between two crystal structures, which is represented by a 3*N*-dimensional vector Δ*X*_*obs *_obtained by superimposing the two structures with minimal Root Mean Squared Deviation (RMSD). An overlap  is calculated for mode *m*, where *V*_*m *_is its eigenvector, |Δ*X*_*obs*_| and |*V*_*m*_| represent the amplitudes of Δ*X*_*obs *_and *V*_*m*_. The mode with the highest overlap is named the dominant mode, if its overlap value is high (|*I*_*m*_| > 0.5).

To assess how collective the observed structural change is, we define the collectivity of Δ*X*_*obs *_as [43], where  and Δ*X*_*obs*, *i *_is the 3D component of Δ*X*_*obs *_at residue *i*.

### 2. Perturbation analysis based on a single dominant mode

To simulate the dynamic effect of a point mutation, we have introduced a perturbation to the local elastic interactions involving a given residue *i *as follows [63]:

(3)

where *δC *represents an arbitrary change to the force constant of those springs connecting residue *i *to its neighbors (residue *j*). This perturbation results in the following change in the Hessian matrix:

(4)

In the single-mode-based SPM proposed earlier [63-65], we analyzed how much the above perturbations change the eigenvalue (or eigenvector) of the mode that dominates the protein functional motions:

(5)

where *δλ*_*m*, *i *_(denoted as *δω*_*m *_in [63]) is the resulting change in the eigenvalue of mode *m*, which is proportional to the elastic energy stored in the springs that connect residue *i *to its neighbors following a displacement of the protein structure in the direction of the eigenvector *V*_*m*_. Because , if we set *δC *= *C*/2, then  (named as normalized *δλ*_*m*, *i*_) gives the fractional contribution of local interactions at residue *i *to mode *m*.  was found to be a good indicator for the dynamic importance of residue positions [63].

### 3. Perturbation analysis based on overall structural fluctuations and structural fluctuations in the direction of observed conformational change

In addition to the perturbation analysis of individual normal modes [63-65], we have also evaluated how much the perturbation in Eq. 3 changes the overall structural fluctuations of the entire protein structure [66] or its active site [67].

The change in the overall structural fluctuations caused by a perturbation at residue *i *(see Eq. 3) is given by the follow score [66]:

(6)

where , *δH*_*i *_is given in Eq. 4, *Tr *represents the trace of a matrix. In practice, *H*^-1 ^can be calculated efficiently by inverting *H *+ *εI *using a sparse linear equation solver (*I *is the identity matrix and *ε *is a small number set to be 0.00001). The purpose of adding a small *ε *is to change the eigenvalues of six zero modes of *H *to positive values so that *H *can be inverted by a linear equation solver. The eigenvectors are not changed by the addition of *ε*. Because *δH*_*i *_is translationally and rotationally invariant, the inclusion of translational and rotational modes in *H*^-1 ^does not affect the calculations in Eq. 6 except for a small increase in the eigenvalues of all non-zero modes by *ε*.

Because , if we assume *δC *= -*C*/2, then δ2, then *δf*_*i*_/⟨(*X *- *X*_0_)^2^⟩ (named as normalized *δf*_*i*_) gives the fractional contribution of local interactions at residue *i *to the overall structural fluctuations of the protein structure. This score was previously used to identify the hinge residues in myosin motor domain [66].

To focus on the fluctuations in the direction of the crystallographically observed conformational change (Δ*X*_*obs*_), we introduce a new fluctuation-based perturbation score:

(7)

Because , if we assume *δC *= -*C*/2, then  (named as normalized ) gives the fractional contribution of local interactions at residue *i *to the fluctuations in the direction of Δ*X*_*obs *_Therefore, those residue positions with high  are significantly involved in the structural fluctuations that sample the new structural state at *X*_0 _+ Δ*X*_*obs *_If the observed conformational change is dominated by a single mode *m *(Δ*X*_*obs*_~*V*_*m*_),  is reduced to . Otherwise,  can be calculated without assuming the existence of a single dominant mode.

### 4. Alternative scores for assessing dynamic importance of residue positions

For comparison with the ENM-based scores of dynamic importance defined in Eqs 5–7, we have also examined the following alternative scores based on structural analysis or GNM modes:

**a**. Two scores based on changes in the pseudo-bond angles and pseudo-dihedral angles associated with the observed conformational change (Δ*X*_*obs*_): they are defined as follows

(8)

where *δθ*(*i *- l, *i*, *i *+ l) is the change in the pseudo-bond angle of three consecutive C_*α *_atoms *i*-1, *i*, *i*+1; *δφ* (*i*-2, *i*-1, *i*, *i*+1) (*δφ* (*i*-1, *i*, *i*+1, *i*+2)) is the change in the pseudo-dihedral angle of four consecutive C_*α *_atoms *i*-2, *i*-1, *i*, *i*+1 (*i*-1, *i*, *i*+1, *i*+2).

**b**. Strain energy score: it is defined as the elastic energy stored in the springs that connect residue *i *to its neighbors following the observed conformational change (Δ*X*_*obs*_):

(9)

**c**. GNM-based fluctuation score: it is defined as follows using the lowest two nonzero GNM modes (following [55])

(10)

where *V*_*mi *_is the *i'th *component of the eigenvector of the GNM mode *m*, and *λ*_*m *_is the corresponding eigenvalue. Note that residues with high  values have low mobility and correspond to hinge centers of a protein structure (see [55]).

### 5. Evaluation of predictions of dynamically important residue positions

Since the dynamically important residues are not completely known for the proteins in our lists, we will evaluate our predictions indirectly by assessing the conservation of the predicted key residues. As we proposed previously [63], the dynamically important residue positions are under functional constraints so they must be either conserved in amino acid type/property, or under co-evolution with each other (See [12]). Therefore, they are expected to be more conserved than those residues not under functional constraints. Consequently, the quality of our predictions can be evaluated by the average conservation scores for the predicted key residue positions.

For a given score of dynamic importance (*S*_*i *_= *δλ*_*m*, *i*_, *δf*_*i*_, , , , , or ), we rank and select the top 15% key residue positions (the choice of a different percentage between 10% and 20% does not qualitatively change our results).

Next we calculate the average conservation score for *N*_*key *_= 15%·N key residue positions (), and for all residue positions (). The conservation scores (*CS*) are calculated based on the multiple sequence alignments of homologous protein sequences by ConSurf server (, [73]). The lowest (highest) conservation score represents the most (least) conserved residue position.

Then we calculate the following Z score to assess statistically how well the predicted residue positions are conserved than average:

(11)

where *σ*_*rand *_is the squared root of the variation of the average conservation score for *N*_*key *_randomly chosen residue positions calculated as follows:



where {*X*_1_, ... } is a randomly selected subset of {*CS*_1_, ... *CS*_*N*_}.

The more negative *Z *is, the more significant is the overall conservation of the key residue positions.

The above evaluation is performed on two lists of test cases (see Table S1&S2 in Additional file 1). For each score, the average (⟨Z⟩) and standard deviation (*σ*_Z_) of the Z scores are calculated for the test cases in both lists. A more negative ⟨Z⟩ is indicative of a better performance

### 6. Generation of a long list of PDB structure pairs

We follow an automated procedure to generate a long list of PDB structure pairs (represented as (pdb1, pdb2)):

**a**. Start from an initial list of 2039 high-resolution protein structures (with sequence identity <30%, resolution <1.6 Å, and R-factor < 0.25) obtained from culledpdb web site ;

**b**. Remove protein structures with <100 residues from the initial list;

**c**. For each protein structure (denoted as pdb1) from the initial list, generate a list of homologous structures (denoted as pdb2) that satisfy the following conditions: (i). sequence identity with pdb1 ≥ 90%; (ii). number of residues ≥ 100; (iii). RMSD between pdb1 and pdb2 ≥ 1 Å. Note: A larger list of homologous structures (denoted as pdb2') with only condition (i) satisfied is compiled to define ligand-binding residues of pdb1, which are either in heavy-atom-contact with a hetero atom in pdb1 or mapped sequentially to residues of a pdb2' which are in heavy-atom-contact with a hetero atom in pdb2';

**d**. Select the pdb2 with the maximal RMSD relative to pdb1 (denoted as pdb2_max_) and add the structure pair (pdb1, pdb2_max_) to the final list.

The final list contains 502 PDB structure pairs. Among them, 473 have ConSurf scores calculated for ≥ 50% of residue positions in pdb1, which are used for Z score calculations. For details see Table S2 in Additional file 1.

## Results and discussion

To evaluate the performance of various scores for selecting dynamically important residues (*δλ*_*m*, *i*_, *δf*_*i*_, , , , , and , see Methods), we have assessed the conservation of the predicted residues (using a Z score, see Methods) for both a short and a long list of test cases (see Table S1&S2 in Additional file 1). The two lists contain a broad range of proteins with diverse biochemical functions (including signaling proteins, DNA-binding proteins, motor proteins and enzymes) and structural architectures (single-domain and multi-domain proteins). The results are summarized in Table 1. Then we have analyzed factors (including ENM parameter, properties of protein structures and conformational changes, see Table 2) that may affect the performance of the ENM-based scores (*δλ*_*m*, *i*_, *δf*_*i*_, ), and their relationships with each other and alternative scores based on structural analysis (, ) and GNM (). Next, we have studied how the predicted key residues correlate with ligand-binding residues and hub residues with high network connectivity, and verified that they form a sparse spatially and sequentially clustered network within protein complexes. Finally, we have illustrated the application of perturbation and correlation protocols to two motor proteins (myosin and kinesin).

**Table 1:** Performance of various scores of dynamic importance assessed by the average (⟨*Z*⟩) and standard deviation (*σ*_*Z*_) of Z scores for two lists of test cases (short list in row 2 and long list in row 3).

	⟨*Z*⟩(*σ*_*Z*_) of						
R_*c*_(Å)			*δf*_*i*_	*δλ*_*m*, *i*_			
7*	**-1.41(2.00)**	**-2.68(2.11)**	**-2.18(2.02)**	**-2.38(2.38)**	**-1.92(2.19)**	**-**0.55(1.30)	0.28(1.27)
7	-1.65(2.09)	-2.63(1.90)	-1.50(1.61)	-2.32(1.84)			
8	-1.92(1.90)	-2.36(1.75)	-1.21(1.82)	-2.07(1.86)			
9	-1.82(2.11)	-2.26(1.83)	-0.61(1.77)	-1.77(2.02)			
10	-1.89(1.94)	-2.32(1.54)	-0.48(1.87)	-1.70(2.07)			
11	-1.90(2.01)	-2.40(1.56)	-0.16(2.11)	-1.55(1.88)			
12	-1.93(2.09)	-2.40(1.67)	-0.09(2.11)	-1.33(2.08)			
13	-1.87(2.11)	-2.24(1.71)	+0.26(2.16)	-1.08(1.97)			
14	-1.93(2.06)	-2.08(1.58)	+0.51(2.30)	-0.96(1.89)			
15	-1.96(1.94)	-2.06(1.52)	+0.79(2.45)	-0.88(1.99)			
7**	-1.20(1.96)	-2.44(1.97)	-2.11(1.82)	-2.21(2.18)			

7*	**-0.06(2.01)**	**-1.80(1.72)**	**-1.81(1.52)**	**-1.60(1.72)**	**-1.98(1.82)**	**-**0.38(1.25)	0.03(1.29)
7	-1.30(1.97)	-1.30(1.97)	-1.02(1.71)	-1.24(1.89)			
8	-0.30(2.27)	-0.97(2.21)	-0.62(1.81)	-0.94(2.00)			
9	-0.36(2.34)	-0.78(2.23)	-0.33(1.87)	-0.65(2.04)			
10	-0.40(2.39)	-0.61(2.30)	-0.08(1.90)	-0.40(2.10)			
11	-0.43(2.44)	-0.51(2.35)	+0.16(2.01)	-0.29(2.20)			
12	-0.49(2.47)	-0.43(2.37)	+0.37(2.09)	-0.23(2.26)			
13	-0.50(2.53)	-0.34(2.39)	+0.54(2.06)	-0.01(2.14)			
14	-0.50(2.56)	-0.24(2.44)	+0.74(2.11)	+0.15(2.16)			
15	-0.47(2.58)	-0.16(2.50)	+0.98(2.20)	+0.32(2.17)			
7**	+0.09(1.88)	-1.68(1.64)	-1.76(1.46)	-1.54(1.66)			

* in combination with heavy-atom-contact

** in combination with heavy-atom-contact, and after removing conserved ligand-binding residues

Note: *σ*_Z_describes the variations of Z scores among the list of test cases. To assess the accuracy of ⟨*Z*⟩, the standard error of ⟨*Z*⟩ for the long list can be estimated as follows:

(473 is the number of protein structure pairs used for Z score calculations, see Methods).

**Table 2:** Dependence of performance of perturbation-based scores on various properties of protein structures and conformational changes evaluated by the average (⟨Z⟩) and standard deviation (*σ*_*Z*_) of the Z scores (statistics of the long list).

Property	Range	⟨Z⟩(*σ*_*Z*_) of		
			*δf*_*i*_	*δλ*_*m*, *i*_
Size	100–153	-1.14(1.34)	-1.30(1.24)	-1.08(1.44)
	153–263	-1.72(1.55)	-1.90(1.41)	-1.32(1.59)
	263–804	-2.48(1.91)	-2.22(1.69)	-2.36(1.81)

RMSD	1.00–1.40	-1.71(1.96)	-1.71(1.58)	-1.58(1.76)
	1.40–2.28	-1.65(1.53)	-1.95(1.48)	-1.62(1.79)
	2.28–20.85	-2.09(1.62)	-1.78(1.48)	-1.66(1.64)

Max overlap	0.05–0.27	-1.61(1.55)	-1.75(1.48)	-1.25(1.58)
	0.27–0.37	-1.85(1.66)	-1.80(1.50)	-1.57(1.79)
	0.37–0.82	-1.93(1.91)	-1.88(1.58)	-1.97(1.71)

Collectivity	0.01–0.13	-1.81(1.68)	-1.97(1.43)	-1.49(1.59)
	0.13–0.33	-1.84(1.66)	-1.85(1.58)	-1.69(1.70)
	0.33–0.79	-1.76(1.82)	-1.61(1.52)	-1.64(1.86)

DynDom domain partition	success	-1.68(1.63)	-1.77(1.45)	-1.47(1.61)
	fail	-2.11(1.88)	-1.92(1.67)	-1.93(1.94)

For the simplicity of evaluation, we have chosen the dynamically important residues as the top 15% residues ranked by a given score. The use of a small percentage like 15% is in line with the previous findings of a sparse network of allosterically coupled residues in various proteins (for example, the allosteric network consists of only 14% of all residues in GPCR, see [13]). It is conceivable that the percentage of dynamically important residues varies from protein to protein. A more sophisticated cutoff scheme will be developed in our future studies.

### 1. Conservation of dynamically important residues

**Figure 1:**
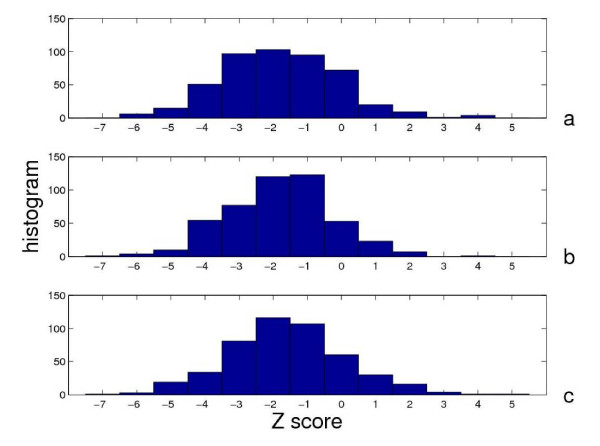
**Distribution of Z scores for 473 cases in the long list using the following three perturbation-based scores**. **(a) **: the percentage of highly conserved (Z ≤ -2) and moderately conserved (-2<Z ≤ -1) cases is 47% and 20%; (b) *δf*_*i*_: the percentage of highly conserved and moderately conserved cases is 48% and 20%; (c) *δλ*_*m*, *i*_: the percentage of highly conserved and moderately conserved cases is 41% and 26%.

We first examine the distribution of Z scores for the key residues predicted by the perturbation-based scores (*δλ*_*m*, *i*_, *δf*_*i*_, , see Figure 1). We have divided all test cases in the long list into three classes based on their Z scores: highly conserved (Z ≥ -2), moderately conserved (-2<Z ≥ -1) and not conserved (-1<Z). Assuming a normal distribution of the average conservation score for *N*_*key *_randomly chosen residue positions (*N*_*key *_is the number of key residues), a Z score ≥ -2 corresponds to 98% confidence level, and a Z score ≥ -1 corresponds to 84% confidence level. Using one of the three scores (*δλ*_*m*, *i*_, *δf*_*i*_, , see Figure 1), the predicted residues in 41–48% (20–26%) of cases are highly (moderately) conserved. Therefore, in most (>67%) of the cases, the dynamically important residues predicted by our perturbation-based scores are either highly or moderately conserved.

We have found large variations in the conservation of dynamically important residues (see Figure 1 and Table 1), which may be attributed to the following causes:

First, it is likely that those key residues co-evolve with each other, which results in weaker conservation of them individually. A detailed analysis of correlated mutations (see [12-14]) is needed to explore this possibility which is beyond the present study.

Second, the accuracy of ENM (with its simplified force field) in predicting the key residues may vary from case to case. We will further explore how the properties of protein structures and conformational changes affect the accuracy in Subsection 3.

Third, some crystallographically observed conformational changes in the long list may not be functionally relevant, therefore the residues involved in these changes are not under functional constraints. The relevance of this factor is supported by the observation that the average Z scores are significantly more negative for the short list (see Table 1) which includes manually selected protein conformational changes whose functional relevance is established in literature.

Judging from the average Z scores (⟨Z⟩),  and *δf*_*i *_perform slightly better than *δλ*_*m*, *i *_(with more negative ⟨Z⟩, see Table 1), which is attributed to the use of more modes than the dominant one in the perturbation and correlation analysis. To show the statistical significance of the above improvement, we perform the following Z score estimation. The standard error of ⟨Z⟩ for the long list is estimated to be  (*σ*_Z _is given in Table 1, 473 is the number of protein structure pairs used for Z score calculations, see Methods). The significance of the difference in ⟨Z⟩ between  and *δλ*_*m*, *i *_is assessed by the following Z score:(-1.8 + 1.60)/*σ*_⟨*Z*⟩ _≈ -2.6 (⟨Z⟩ values are given in Table 1). This large negative Z score indicates that the improvement from *δλ*_*m*, *i *_to  is statistically significant. Similarly, we can show that the improvement from *δλ*_*m*, *i *_to *δf*_*i *_is also statistically significant.

### 2. Optimal performance of perturbation and correlation analysis at a low cutoff distance *R*_*c *_= 7 Å

To explore how the performance of perturbation-based scores (*δλ*_*m*, *i*_, *δf*_*i*_, ) depends on ENM parameter (cutoff distance *R*_*c*_), the Z score calculations are done for a range of *R*_*c *_values (7 Å ≤ *R*_*c *_≤ 15 Å) (we do not consider *R*_*c*_<7 Å because it causes many zero modes to arise due to insufficient connectivity in ENM). For both the short and long list, we have found that the best performance (the most negative ⟨Z⟩) for *δλ*_*m*, *i*_, *δf*_*i *_and  is attained at *R*_*c *_= 7 Å (see Table 1). In addition, if the C_*α*_-C_*α *_distance cutoff at 7 Å is combined with a heavy-atom-contact criterion (namely, two residues are in contact if the minimal distance between their heavy atoms is <4 Å), the performance is further improved (see Table 1).

The above results suggest that the accuracy of ENM-based perturbation and correlation analysis is optimal when the range of C_*α*_-C_*α *_interactions in ENM matches the center of the distribution of C_*α*_-C_*α *_distances between residues in atomic contacts (4.4 Å ~12.8 Å, see [74]). This is contrary to previous studies that found better ENM performance for fitting crystallographic B factors at relatively high *R*_*c *_(15 Å ≤ *R*_*c *_≤ 24 Å) [62]. This is also at odds with our finding that the maximal overlap for the dominant mode is lower at *R*_*c *_= 7 Å (0.35 ± 0.14 for the long list) than at 8 Å ≤ *R*_*c *_≤ 11 Å (0.42 ± 0.16 for the long list). Interestingly, the finding of optimality at low *R*_*c *_is roughly consistent with a recent study that found the optimal descriptions of structural fluctuations and conformational changes by a generalized anisotropic network model at *R*_*c *_= 8 Å [75].

The above discrepancy may be explained as follows. Intuitively, the use of high *R*_*c *_tends to suppress local motions (for example, in a dangling loop) that often arise as extra zero modes at low *R*_*c*_. Therefore, high *R*_*c *_helps to remove the tip effects (overly flexible pieces of proteins that protrude out of the main globular body, see [76]) and improve the description of collective domain motions observed crystallographically [11]. However, the introduction of additional elastic interactions beyond the range of physical interactions between contacting residues may compromise the accuracy of modeling local perturbations by point mutations, which explains the lower performance as *R*_*c *_increases from 7 Å to 15 Å (Table 1). Therefore, the optimal choice of ENM parameter is application-dependent. To simulate the dynamic effects of local perturbations to protein structures, it is preferable to use a relatively low *R*_*c*_, which allows the structural fluctuations and low-frequency modes of ENM to respond more sensitively to residue-position-specific perturbations to local interactions (see Eq. 3 of Methods). Indeed, we have found that the percentage of structural fluctuations contributed by the top 15% key residues (ranked by *δf*_*i*_) decreases significantly (from 59 ± 10% to 31 ± 18%, for cases in the long list) as *R*_*c *_increases from 7 Å to 15 Å. Therefore, despite the findings that low-frequency modes are robust to ENM parameters (see [77]), the use of perturbation methods to probe ENM dynamics [63,64,53,78] remains feasible (especially at low *R*_*c*_). In this study, we will use ENM constructed with *R*_*c *_= 7 Å and the heavy-atom-contact criterion.

### 3. Dependence of performance on properties of protein structures and conformational changes

We now study how the performance of three perturbation-based scores (, *δf*_*i*_, *δλ*_*m*, *i*_) depends on four properties of protein structures and conformational changes in the long list. To reduce the statistical noise due to large variations in Z scores (large width in distribution of Z scores, see Figure 1), we have performed the following three-tier averaging: for each property, all test cases are sorted by the property value and then divided equally into three tiers (the bottom 1/3, middle 1/3 and top 1/3 go into the low, medium and high tier, respectively); the average (*σ*_*Z*_) and the standard deviation (⟨Z⟩) of the Z scores are calculated separately for each tier (see Table 2). The statistical significance of the observed differences in ⟨Z⟩ between tiers can be demonstrated following the same Z score estimation as given at the end of subsection 1.

#### a. Protein size (number of residues in a protein structure)

For all three scores (, *δf*_*i*_, *δλ*_*m*, *i*_), ⟨Z⟩ decreases significantly from low-size, medium-size to high-size tier. Thus a better performance is expected for larger proteins despite large variations in Z scores. This result agrees with the general understanding that ENM modeling is more suitable for large proteins whose low-frequency dynamics is more dependent on the global shape, and less sensitive to inaccuracy in local interactions.

#### b. RMSD between two PDB structures

The dependence on RMSD differs between three scores – *δλ*_*m*, *i *_has the weakest RMSD-dependence; *δf*_*i *_performs best in medium-RMSD tier;  performs best in high-RMSD tier. Therefore, *δf*_*i *_and  may be used selectively depending on the magnitude of conformational change. It is encouraging that  is applicable to large-scale conformational changes beyond thermal fluctuations.

#### c. Overlap of the dominant mode for the observed conformational change

A negative correlation (⟨Z⟩ decreases from low-, medium- to high-overlap tier) is found for all three scores, which is strongest for *δλ*_*m*, *i *_and relatively weak for *δf*_*i *_and . This is expected because *δλ*_*m*, *i *_relies on the assumption of a single dominant mode while *δf*_*i *_and  do not. Therefore ENM remains valid for describing structural fluctuations and residues involved even if it fails to capture the observed conformational changes by a single dominant mode.

#### d. Collective nature of the observed conformational change

Previous studies have found that ENM performs better in describing protein conformational changes (see Yang 2007) with high collectivity (for definition, see Methods). We have explored the dependence of Z scores on the collectivity of the observed conformational change. Surprisingly, it is found that high/medium collectivity only improves the performance of *δλ*_*m*, *i *_but not  or *δf*_*i*_. Therefore, unlike other ENM assessing metrics (such as the overlap of the dominant mode, see [48]), the accuracy of ENM-based predictions for dynamically important residues (using  or *δf*_*i*_) does not strongly depend on the collectivity of conformational changes.

In complement to the collectivity calculations, we have also determined how the performance depends on whether the observed conformational change can be approximated by rigid-body domain motions. To this end, we have applied DynDom [28] to the observed conformational changes in the long list, which are then divided into two subsets (success/failure) depending on whether DynDom succeeds/fails in dynamic domain partition. Domain partitions were made successfully by DynDom for 135 out of 502 cases. We have found that all three scores perform better if the conformational change involves domain motions. For those cases where domain partition succeeds,  performs the best; for those cases where domain partition fails, *δf*_*i *_performs the best.

In sum, the performance of the three perturbation-based scores depends more on the protein size than the properties of conformational changes (see Table 2). In particular, *δf*_*i *_and  perform well even when the observed conformational change is not collective or not dominated by a single mode. In fact, they outperform *δλ*_*m*, *i *_under those conditions (see Table 2). Therefore the fluctuation-based perturbation and correlation analysis may be applied more broadly than the single-mode-based protocol to cases where many modes are involved in the functional motions.

### 4. Relationship between various scores of dynamic importance

To evaluate how much the key residues predicted by two scores of dynamic importance (denoted as S1 and S2) overlap with each other, we have calculated a correlation factor – it is defined as the enrichment in the probability of finding a key residue predicted by S2 within the set of key residues predicted by S1 relative to that expected if randomly selecting a residue from all residues in a protein. In practice, the correlation factor is calculated as , where *N*_*key*12 _is the number of key residues predicted by both S1 and S2, *N*_*key*1_(*N*_*key*2_) is the number of key residues predicted by S1 (S2), and *N *is the total number of residues. A correlation factor >>1 indicates strong correlation between S1 and S2. Based on the statistics of the long list (same below in this subsection), the correlation factor between the key residues predicted by  and *δf*_*i *_(*δλ*_*m*, *i*_) is 3.57 ± 1.13 (3.18 ± 1.39). In addition, 55 ± 17% (49 ± 21%) of the key residues predicted by  and *δf*_*i*_(*δλ*_*m*, *i*_) overlap with each other. Namely, 55 ± 17% (49 ± 21%) of the key residues predicted by  are also predicted by *δf*_*i*_(*δλ*_*m*, *i*_). Therefore, there are strong overlaps between the key residues predicted by the three perturbation-based scores (, *δf*_*i *_and *δλ*_*m*, *i*_). Many common residues are involved in both the equilibrium fluctuations (in all directions as probed by *δf*_*i*_) and the large conformational change (in a particular direction as probed by ). Therefore, it may be possible to predict those residues involved in the *slow *conformational change from a known structural state toward an unknown structural state, by simulating the *fast *equilibrium fluctuations near the known state [27]. This useful strategy avoids the need for costly long-time MD simulations.

Compared with the above perturbation-based scores, the residues involved in the observed conformational changes as identified by alternative scores based on energetic or structural analysis (, , , see Methods) are much less conserved (see Table 1). Therefore, we infer that not all residues involved in the observed conformational changes are equally constrained by functions. From the point of view of structural transition, not all changes in local structures/interactions are equally important to the transition – those early-occurring changes are more likely to affect the transition rate than the late-occurring ones (see [60]). It is conceivable that the early structural changes involve intrinsic motions described by the low-frequency normal modes. Therefore, by capturing these early structural changes, the NMA-based perturbation and correlation analysis can predict dynamically important residues more accurately than standard structural and energetic analysis.

Following [55] we have also calculated a GNM-based score for predicting hinge residues with minimal mobility. This score () is based on the calculation of mean-squared fluctuations due to the lowest two GNM modes (see Methods). It is found that the hinge residues (defined as the top 15% residues ranked by ) are significantly conserved (see Table 1), which supports their functional importance [55]. Nevertheless, they do not overlap significantly with the key residues predicted by the perturbation and correlation analysis. Indeed, the correlation factor between the key residues predicted by  and  is only 1.6 ± 0.95, and only 25 ± 14% of key residues predicted by  and  overlap with each other. Such a lack of correlation is not unexpected, because these methods are based on different principles: the GNM score depends on the distribution of structural fluctuations, while  and *δf*_*i *_probe how the overall fluctuations are coupled to local perturbations. Therefore, the two methods may complement each other in probing different aspects of protein conformational dynamics.

### 5. Correlation between dynamically important residues and ligand-binding residues

The correlation between conformational dynamics and ligand binding in enzymes has been explored computationally in several recent studies. In one study, Yang and Bahar found that catalytic residues are often co-localized with hinge residues identified by GNM [55]. Another study found that the ability to trigger large changes in conformational distribution makes a good score function for predicting ligand-binding site [52,53]. One can argue that evolution favors ligand binding to dynamically important regions of proteins to effectively trigger or regulate protein dynamics. Thus these key residues may be constrained by both conformational dynamics and ligand binding affinity.

To explore the correlation between ligand-binding residues and the dynamically important residues predicted by our perturbation and correlation analysis, we have evaluated if these two sets of residues co-localize in protein structures. To enable analysis of a large dataset, the identification of ligand-binding residues is automated using two criteria: first, they are highly conserved (with grade = 9 based on the ConSurf conservation scores, [73]); second, they are in heavy-atom-contact with at least one hetero atom (from the HETATM records of PDB files, excluding waters and hydrogen atoms) in the PDB file of the given structure or a homologous protein structure with ≥ 90% sequence identity. The first criterion allows us to automatically filter out those unconserved residues that bind biologically irrelevant ligands or reagents used for crystallization (see [79]). For each test case of the long list, we have calculated a correlation factor (defined as the enrichment in the probability of finding a ligand-binding residue from the set of key residues relative to that expected if randomly selecting a residue from all residues in a protein). A high correlation factor (>>1) indicates a high level of co-localization between the two residue sets. The average correlation factors for the three perturbation-based scores (, *δf*_*i *_and *δλ*_*m*, *i*_) are 1.58 ± 1.32, 1.45 ± 1.38 and 1.47 ± 1.30, respectively. This result indicates a weak tendency of co-localization – there is ~1.5 times higher probability for ligands to bind with dynamically important residues than random. The ligand-binding residues only comprise a small fraction of the predicted key residues (the average percentage of key residues which are also ligand-binding is only ~6–7%). Therefore, we infer that the conservation of the predicted key residues is not primarily due to the conservation of ligand-binding residues. Indeed, we have reevaluated the performance of the perturbation-based scores after removing all highly conserved ligand-binding residues, the average Z scores are only slightly reduced (see Table 1). Therefore, most of the predicted key residues are evolutionarily constrained by their roles in conformational dynamics rather than specific interactions with ligands.

### 6. Correlation between dynamically important residues and hub residues

In an ENM, the hub residues with high connectivity (defined as the number of neighboring residues in heavy-atom-contact with a given residue) are likely involved in both structural stability and dynamics [80]. Indeed, we have found a high level of conservation for the hub residues with top 15% connectivity (their Z scores are -3.35 ± 1.42 for cases in the long list). Because the residue-position-specific perturbation (see Eq. 3 of Methods) is applied to the interactions between a given residue and its neighbors, it is natural to expect such perturbation to be larger for residues with more neighbors than those with fewer neighbors. To evaluate the connectivity-dependence of the perturbation-based scores, we have assessed how much the predicted key residues overlap with the hub residues.

The correlation factors for the three perturbation-based scores (, *δf*_*i *_and *δλ*_*m*, *i*_) are 1.65 ± 0.45, 1.69 ± 0.48 and 1.65 ± 0.50, respectively (statistics of long list). This result indicates a relatively weak overlap between the two sets of residues – there is ~1.7 times higher probability to find dynamically important residues to be hub residues than random. The hub residues only comprise a minor fraction of the predicted key residues (the average percentage of key residues predicted by the three scores which are also hub residues is 34–35% for cases in the long list). Thus the majority (>65%) of the dynamically important residues are not hub residues, whose dynamic importance is not due to their high connectivity in ENM.

### 7. A sparse network formed by dynamically important residues

The three perturbation-based scores (after normalization, see Methods) give the fractional contribution of each residue position to either structural fluctuations (, *δf*_*i*_) or elastic energy (*δλ*_*m*, *i*_). For cases in the long list, on average 59–70% of the structural fluctuations or elastic energy are contributed by the top 15% key residues predicted by one of the three scores. Therefore the protein dynamics is dominated by a sparse set of key residues, which agrees with similar findings by sequence-based analysis (see [12,13]).

To further explore how the key residues are distributed in 3D space and along protein sequence, we have calculated a spatial (or sequential) correlation factor defined as the enrichment in the probability of finding two key residues in heavy-atom-contact (or sequentially separated by <10 residues) relative to that expected if these residues are distributed randomly in a protein structure (or sequence). In practice, the spatial correlation factor is calculated as , where *N*_*key*, *par*_(*N*_*par*_) is the number of key residue pairs (all residue pairs) that are in heavy-atom-contact. For cases in the long list, the spatial correlation factors for the three scores (, *δf*_*i *_and *δλ*_*m*, *i*_) are 3.69 ± 0.63, 3.61 ± 0.59 and 3.56 ± 0.69, respectively; the sequential correlation factors for the three scores are 2.14 ± 0.67, 1.99 ± 0.58 and 2.08 ± 0.66, respectively. Therefore, the dynamically important residues are clustered both in 3D space and along protein sequence (though to a less extent), which form a strongly coupled network. This finding complements a recent finding that the residues involved in local motions of allosteric proteins are correlated in 3D space and along sequence [24]. The high degree of spatial and sequential connectivity allows these key residues to mediate signal transmissions over long distances between spatially separated functional sites.

### 8. Case studies of dynamically important residues in myosin and kinesin

Finally, we will apply the above methods to the functional conformational changes in myosin and kinesin – two filament-based motor proteins driven by ATP binding and hydrolysis. Myosin has been previously studied by the single-mode-based perturbation analysis [64] and correlation analysis [66]. Here we will focus on a comparison of the predictions of dynamically important residues made by various protocols (, *δf*_*i*_, *δλ*_*m*, *i*_, , connectivity and DynDom).

#### a. myosin

**Figure 2:**
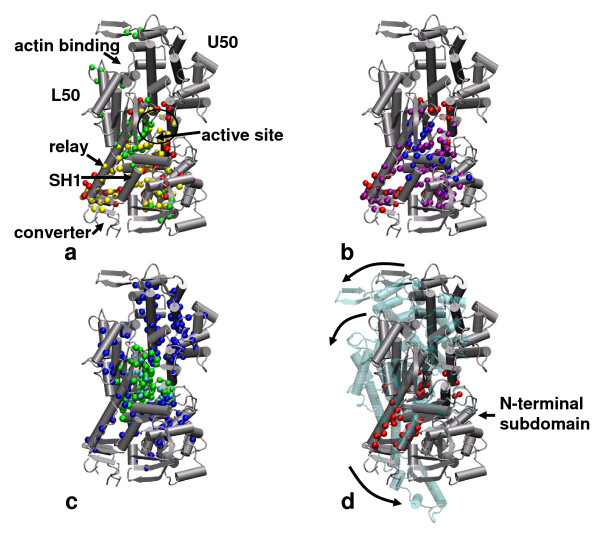
**Comparison of dynamically important residues in myosin predicted by various protocols**. **(a)**. key residues predicted by  and *δf*_*i *_are shown as red and green spheres, the common residues are colored in yellow, key components and sites in myosin are also labeled; (b). key residues predicted by  and *δλ*_*m*, *i *_are shown as red and blue spheres, the common residues are colored in purple; (c). key residues predicted by GNM score and high connectivity are shown as green and blue spheres, the overlapping residues are colored in cyan; (d). hinge residues identified by DynDom are shown as red spheres. Also shown in panel (d) is the observed conformational change from a pre-powerstroke myosin structure (PDB: 1VOM, colored in silver) to a post-powerstroke myosin structure (PDB: 2AKA, colored in cyan) superimposed along the N-terminal subdomain (residues 80–186), including rotations of U50, L50 and converter subdomains (shown as arrows). For details of the predicted key residues, see Table S3 in Additional file 1.

We have examined the large conformational change (see Figure 2d) from a pre-powerstroke transition-state structure (PDB: 1VOM) to a post-powerstroke nucleotide-free structure (PDB: 2AKA), which pertains to the force generation and hydrolysis product release in myosin [81]. This conformational change involves large rotations of the converter, the upper/lower 50 kDa subdomains (U50/L50) relative to the N-terminal subdomain (see Figure 2d). This collective conformational change is captured by the lowest two ENM modes calculated for 1VOM[66]. The key residues predicted by , *δf*_*i *_and *δλ*_*m*, *i *_are highly conserved (with Z scores of -8.35, -7.80, -7.70, respectively). The key residues predicted by  overlap strongly with those predicted by *δf*_*i*_(66% identical, see Figure 2a) and *δλ*_*m*, *i *_(74% identical, see Figure 2b). These residues are clustered in 3D space, forming a sparse network that connects the active site to the force-generating component (converter) via two flexible connectors – relay helix and SH1 helix (see Figure 2a, b). Interestingly, a few key residues predicted by *δf*_*i *_are located near the actin-binding cleft (see Figure 2a), which may allow actin binding to regulate the structural fluctuations of myosin [66].

For comparison, we have also shown the hinge residues predicted by the GNM score [55], the hub residues with high connectivity (see Figure 2c) and the hinge-bending residues identified by DynDom (see Figure 2d). They overlap insignificantly with the key residues predicted by  (18% identical for the GNM score, 27% identical for the hub residues, 11% identical for DynDom). Unlike the key residues predicted by the perturbation-based scores, the hub residues are extensively distributed rather than clustered in space, while the hinge residues predicted by the GNM score are concentrated near the active site [55]. Therefore, they do not provide a signaling path that connects the active site to converter.

#### b. kinesin

**Figure 3:**
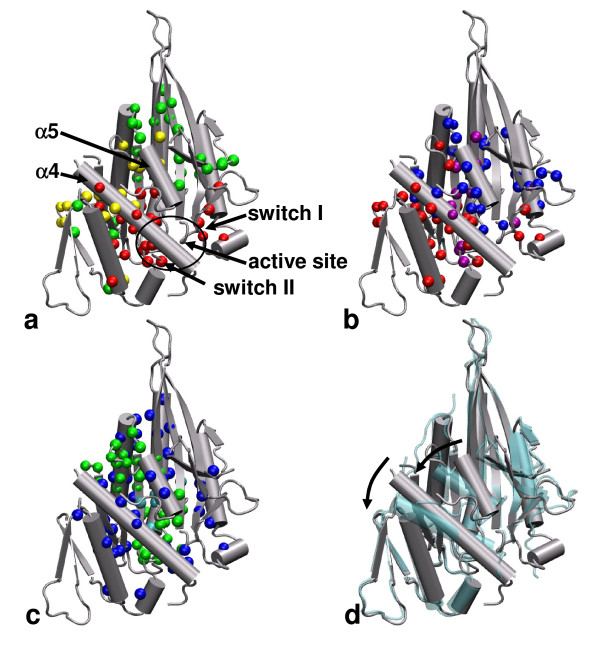
**Comparison of dynamically important residues in kinesin predicted by various protocols**. **(a)**. key residues predicted by  and *δf*_*i *_are shown as red and green spheres, the common residues are colored in yellow, key components and sites in kinesin are also labeled; (b). key residues predicted by  and *δλ*_*m*, *i *_are shown as red and blue spheres, the common residues are colored in purple; (c). key residues predicted by GNM score and high connectivity are shown as green and blue spheres, the overlapping residues are colored in cyan. Shown in panel (d) is the observed conformational change from an ADP-bound KIF1A structure (PDB: 1I5S, colored in silver) to an ATP-analog-bound KIF1A structure (PDB: 1VFW, colored in cyan), including rotations and translations of *α*4, *α*5 helices (shown as arrows). For details of the predicted key residues, see Table S4 in Additional file 1.

We have studied the observed conformational change (see Figure 3d) from an ADP-bound KIF1A structure (PDB: 1I5S) to an ATP-analog-bound KIF1A structure (PDB: 1VFW) which pertains to ADP release followed by ATP-binding-induced force generation in kinesin [82,83]. This conformational change involves an *en block *translation and rotation of the switch II cluster (including *α*4, *α*5 helices and switch II at the active site, see Figure 3a, d) and local changes in the switch I region (see Figure 3a, d) [82,83]. The key residues predicted by , *δf*_*i *_and *δλ*_*m*, *i *_are highly conserved (with Z scores of -4.48, -2.09, -5.21, respectively). The key residues predicted by  overlap moderately with those predicted by *δf*_*i*_(44% identical, see Figure 3a) and *δλ*_*m*, *i *_(30% identical, see Figure 3b). The divergence among the three perturbation-based scores may be due to the involvement of many modes in the structural fluctuations and the observed conformational changes. However, despite the lack of a dominant mode (the maximal overlap is only 0.23), the perturbation-based scores still predict a highly conserved network of residues that connect the active site to the force-generating neck linker via *α*4 helix (see Figure 3a, b, d).

For comparison, we have also shown the hinge residues predicted by the GNM score, and the hub residues with high connectivity (see Figure 3c). They overlap moderately with the key residues predicted by (30% identical for the GNM score, 22% identical for the hub residues). Similar to myosin, the hub residues are extensively distributed while the hinge residues predicted by the GNM score are clustered spatially (see Figure 3c).

## Conclusion

In this study, we have performed a large-scale evaluation of the predictions of dynamically important residues by a variety of computational protocols including three based on the perturbation and correlation analysis of ENM, and alternative ones based on structural analysis or GNM. We have found that the two fluctuation-based scores ( and *δf*_*i*_), by accounting for contributions from many ENM modes, outperform the previously proposed single-mode-based score (*δλ*_*m*, *i*_, see [63-65]) especially in cases where many modes are involved in the conformational changes. The dynamically important residues predicted by the perturbation-based scores (, *δf*_*i*_, *δλ*_*m*, *i*_) are strongly or moderately conserved, and they form a sparse network of residues which are clustered both in 3D space and along protein sequence. Their overall conservation is attributed to their dynamic role rather than ligand binding or high connectivity in ENM. Future functional studies are needed to dissect the detailed roles of these predicted residues.

As shown by numerous studies (see reviews [49-51]), the coarse-grained ENM captures the essence of collective protein dynamics, which is largely determined by the global shape of and local packing in protein structures. Therefore, ENM provides a simple and adequate framework for exploring, with a residue level of details, how much a perturbation to local packing affects protein structural dynamics. Nevertheless, our perturbation methods are only useful for locating the residue positions of dynamic importance, but not for predicting the functional effects of a particular perturbation, which may require more accurate modeling of energetics and dynamics beyond coarse-grained modeling.

Compared with standard NMA, the major advantage of the fluctuation-based scores ( and *δf*_*i*_) is their low computational cost (the CPU time for analyzing the long list of >500 pairs of structures is < 25 minutes on a quad-core Xeon workstation). The calculation does not require the solution of all ENM modes which is computationally expensive and memory-demanding for large proteins. Instead, it only requires the inversion of a sparse Hessian matrix (see Eq. 6 and 7 in Methods), which can be done much faster than NMA, thanks to the availability of highly efficient sparse linear equation solver [84]. The sparseness of the Hessian matrix is particularly high for low *R*_*c *_where the perturbation and correlation analysis is optimal (see Table 1).

The perturbation-based protocols are different from and complementary to the GNM-based technique (see [55]) and various others (see [56] and references therein) that find hinge regions of protein domain motions. The dynamically important residues defined by our perturbation analysis are different from the hinge residues defined based on other criteria (see [56]). Our approach can be applied to protein conformational changes that are not rigid-body domain motions, where inter-domain hinges are not well defined.

The finding of conservation of the key residues involved in the fluctuations toward the direction of a structural transition supports the functional importance of such fluctuations. For a biochemical transition from an apo to a ligand-bound state, our finding supports the proposal that sampling of the ligand-bound-state conformation in the absence of a ligand is essential for the transition (see [4]). The key residues predicted by  are likely involved in coordinating such pre-existing sampling, which call for future mutational and functional studies of these residues.

It is increasingly recognized that in many proteins the allosteric effects on function involve changes in dynamics in the absence of detectable structural changes [4,5,85]. The ENM-based perturbation and correlation analysis involves local perturbations that do not change the equilibrium structure, so they are well suited for simulating the allosteric effects via dynamic regulation and entropic changes [85]. These methods complement alternative computational techniques that simulate changes in equilibrium structure during biomolecular transitions, including several transition pathway modeling methods [57-61]. Our perturbation methods also complement alternative allostery-modeling techniques, including those based on Markov propagation of information across the protein structure [86-88].

The perturbation methods described here are similar in spirit to the dynamics perturbation analysis algorithm proposed by Ming and Wall [89]. Both are based on the first order perturbation theory, although the mathematic form of the perturbation to Hessian matrix differs.

Besides predicting dynamically important residues, our perturbation methods can also be used to parameterize ENM to fit crystallographic temperature factors, which depend on both the intrinsic dynamics of a protein structure and its crystalline environment (see [90]). We have also explored the use of perturbation and correlation analysis to probe allosteric couplings in F_1 _ATPase [91] and the coupling between normal modes in myosin motor [92].

## Authors' contributions

WZ conceived of the study, implemented and tested the methods, and drafted the paper. MT provided assistance with webserver applications and references preparation. Both authors read and approved the final manuscript.

## Supplementary Material

Additional file 1 **Supplementary tables**. The data provided offer detailed information for the two lists of test cases (Tables S1&S2) and the predicted key residues in myosin and kinesin (Tables S3&S4). Click here for file
