# Supplementary material for: Large-scale evaluation of dynamically important residues in proteins predicted by the perturbation analysis of a coarse-grained elastic model
Source: BMC Struct Biol. 2009 Jul 10;9:45. doi: 10.1186/1472-6807-9-45 (PMC2719638; doi:10.1186/1472-6807-9-45)
Supplement: Additional file 1 — Supplementary tables. The data provided offer detailed information for the two lists of test cases (Tables S1&S2) and the predicted key residues in myosin and kinesin (Tables S3&S4). [file 1472-6807-9-45-S1.doc]

**Supplementary Tables**

**Table S1**. The short list of 25 protein structure pairs: length1 and length2 are the number of residues in the two protein structures (PDB1 and PDB2). RMSD is the RMSD between PDB1 and PDB2.

| PDB1 PDB2 length1 length2 RMSD CONSURF Protein Name  (Å) |
| --- |
| 1heiA 1a1vA 443 429 1.1 Yes HCV HELICASE  1aonH 1aonA 524 524 12.3 Yes GROEL  1bmfE 1bmfF 466 466 3.8 Yes BOVINE MITOCHONDRIAL F1-ATPASE  1bp5A 1a8eA 328 329 6.7 Yes SERUM TRANSFERRIN  1bpxA 1bpyA 331 326 2.8 Yes DNA POLYMERASE BETA  1cllA 1cfdA 72 75 4.6 Yes CALMODULIN  1ex6A 1ex7A 186 186 3.6 Yes GUANYLATE KINASE  1hhpA 1ajxA 99 99 1.0 Yes UNLIGANDED HIV-1 PROTEASE  1hilA 1himH 211 211 1.2 Yes IGG2A-KAPPA 17/9 FAB (LIGHT CHAIN)  1i5sA 1vfwA 330 329 2.2 Yes KINESIN-LIKE PROTEIN KIF1A  1ih7A 1ig9A 897 901 6.5 Yes DNA POLYMERASE  1lfhA 1lfgA 691 691 6.4 Yes LACTOFERRIN  1ompA 1anfA 370 370 3.8 Yes D-MALTODEXTRIN BINDING PROTEIN  1rkmA 2rkmA 517 517 3.1 Yes OLIGO-PEPTIDE BINDING PROTEIN  1tagA 1tndA 314 323 1.2 Yes TRANSDUCIN-ALPHA  1vomA 2akaA 730 764 6.6 Yes MYOSIN  2ktqA 3ktqA 528 539 2.0 Yes LARGE FRAGMENT OF DNA POLYMERASE I  2laoA 1lstA 238 238 4.7 Yes LYSINE, ARGININE, ORNITHINE-BINDING PROTEIN  3chyA 1chnA 128 126 1.4 Yes CHEY  3tglA 4tglA 265 265 1.6 Yes TRIACYL-GLYCEROL ACYLHYDROLASE  4akeA 1ankA 214 214 7.1 Yes ADENYLATE KINASE  4q21A 5p21A 169 166 1.6 Yes C-H-RAS P21 PROTEIN CATALYTIC DOMAIN  5cscA 6cscA 429 437 2.4 Yes CITRATE SYNTHASE  8adhA 6adhA 374 374 1.4 Yes APO-LIVER ALCOHOL DEHYDROGENASE  9aatA 1amaA 401 401 1.7 Yes ASPARTATE AMINOTRANSFERASE |

**Table S2**. The long list of 502 protein structure pairs: length1 and length2 are the number of residues in the two protein structures (PDB1 and PDB2). RMSD is the RMSD between PDB1 and PDB2.

| PDB1 PDB2 length1 length2 RMSD CONSURF Protein Name  (Å) |
| --- |
| 1io7A 1f4uB 366 366 2.2 Yes CYTOCHROME P450 CYP119  2qudA 1dwnB 121 127 1.1 No Coat protein  2h6fB 1d8eB 410 407 1.6 Yes Protein farnesyltransferase beta subunit  1x1zA 1lolB 215 214 1.3 Yes Orotidine 5'-phosphate decarboxylase  2oqgA 2oqgD 106 105 1.6 Yes Possible transcriptional regulator, ArsR family protein  2fp1A 2fp1B 165 164 2.3 Yes Chorismate mutase  2olrA 1oenA 535 524 2.7 Yes Phosphoenolpyruvate carboxykinase  1o26A 1kq4D 217 206 2.0 Yes Thymidylate synthase thyX  1kmvA 1yhoA 186 186 1.3 Yes DIHYDROFOLATE REDUCTASE  1ra9A 1rg7A 159 159 1.8 Yes DIHYDROFOLATE REDUCTASE  1e9gA 1pypB 284 281 1.6 Yes INORGANIC PYROPHOSPHATASE  2pyqA 2pyqD 107 107 1.2 Yes Uncharacterized protein  1mg4A 1uf0A 101 116 1.7 Yes DOUBLECORTIN-LIKE KINASE (N-TERMINAL DOMAIN)  1i58A 1i59B 189 188 2.9 Yes CHEMOTAXIS PROTEIN CHEA  2rb7A 2rb7B 345 345 1.2 Yes Peptidase, M20/M25/M40 family  2heuA 2i58A 390 385 2.7 Yes Sugar ABC transporter, sugar-binding protein  2gb4A 3bgiB 231 231 1.1 Yes Thiopurine S-methyltransferase  2plrA 2plrB 211 207 2.0 Yes Probable thymidylate kinase  2iwrA 2bmjA 174 174 1.4 Yes CENTAURIN GAMMA 1  1eaqA 1cmoA 121 127 4.8 Yes RUNT-RELATED TRANSCRIPTION FACTOR 1  2dkoB 1i3oD 103 101 3.5 Yes Caspase-3  2eb4A 2eb6C 261 267 1.2 Yes 2-oxo-hept-3-ene-1,7-dioate hydratase  2nxwA 2q5oB 530 530 2.0 Yes PHENYL-3-PYRUVATE DECARBOXYLASE  2de3A 2de2A 344 347 2.1 Yes DIBENZOTHIOPHENE DESULFURIZATION ENZYME B  3b50A 2cexD 310 305 3.5 Yes Sialic acid-binding periplasmic protein siaP  3chbD 1g8zF 103 103 1.1 No CHOLERA TOXIN  2pefA 2peeB 366 387 7.8 Yes Serine protease inhibitor  2zgwA 2dz9B 235 235 1.4 Yes biotin--[acetyl-CoA-carboxylase] ligase  1xodA 1tj6A 106 115 1.6 Yes Spred1  1oboA 1dx9A 169 168 1.0 Yes FLAVODOXIN  1kqrA 1kriA 160 160 1.4 Yes VP4  1dbwA 1d5wC 123 121 1.4 Yes TRANSCRIPTIONAL REGULATORY PROTEIN FIXJ  1k0mA 1rk4B 235 206 7.5 Yes CHLORIDE INTRACELLULAR CHANNEL PROTEIN 1  2nrlA 1mytA 145 146 1.0 Yes Myoglobin  2haiA 2dxsB 560 517 1.4 Yes HEPATITIS C VIRUS NS5B RNA POLYMERASE  1m15A 1m80A 356 344 2.9 Yes arginine kinase  1g8aA 2nnwB 227 227 1.5 Yes FIBRILLARIN-LIKE PRE-RRNA PROCESSING PROTEIN  2nl9A 1wsxA 140 162 1.9 Yes FUSION PROTEIN CONSISTING OF Induced myeloid leukemia cell differentiation protein Mcl-1 homolog  2pvbA 3palA 107 107 1.2 Yes PARVALBUMIN  2etxA 2azmB 194 193 1.5 Yes Mediator of DNA damage checkpoint protein 1  2j6iA 2fssB 353 346 1.1 Yes FORMATE DEHYDROGENASE  3f9xA 3f9xC 160 160 1.1 Yes Histone-lysine N-methyltransferase SETD8  1f7lA 1f80A 118 118 1.5 Yes HOLO-(ACYL CARRIER PROTEIN) SYNTHASE  2hqsA 1crzA 412 397 2.5 Yes Protein tolB  3bmzA 2zf3F 185 181 1.9 No Putative uncharacterized protein  2c4jA 1hncD 217 217 1.8 Yes GLUTATHIONE S-TRANSFERASE MU 2  1jbeA 1djmA 126 129 2.3 Yes Chemotaxis protein CHEY  3bonA 2iseB 416 421 2.9 Yes Neurotoxin A  3etjA 1b6rA 355 349 1.4 Yes Phosphoribosylaminoimidazole carboxylase ATPase subunit  1qopB 2dh6A 390 331 3.5 Yes TRYPTOPHAN SYNTHASE BETA CHAIN  2driA 1ba2A 271 271 6.2 Yes D-RIBOSE-BINDING PROTEIN  2q3wA 1sjgA 109 112 2.7 Yes Toluene-4-monooxygenase system ferredoxin subunit  2gdgA 1fimA 114 102 1.2 Yes Macrophage migration inhibitory factor  1wn2A 2d3kB 118 118 1.2 Yes Peptidyl-tRNA hydrolase  1oh0A 1w01A 125 125 1.1 Yes STEROID DELTA-ISOMERASE  1k5nA 1m05C 276 269 1.6 Yes major histocompatibility complex molecule HLA-B*2709  1h99A 1tlvA 220 204 12.6 Yes TRANSCRIPTION ANTITERMINATOR LICT  1a4iA 1digB 285 295 1.4 Yes METHYLENETETRAHYDROFOLATE DEHYDROGENASE / METHENYLTETRAHYDROFOLATE CYCLOHYDROLASE  3dtbA 2gmvB 620 602 1.4 Yes Phosphoenolpyruvate carboxykinase, cytosolic [GTP]  1d8wA 1de5D 394 416 1.0 Yes L-RHAMNOSE ISOMERASE  1y9lA 2jw1A 110 115 2.9 No Lipoprotein mxiM  2hq2A 1u9tA 330 327 1.7 Yes Putative heme/hemoglobin transport protein  1sx5A 1rveB 244 244 1.9 No Type II restriction enzyme EcoRV  1nytA 1nytC 271 269 1.6 Yes Shikimate 5-dehydrogenase  1nu0A 1ovqA 127 138 6.1 Yes Hypothetical protein yqgF  1eyhA 1inzA 144 144 6.0 Yes EPSIN  1iujA 1iujB 102 103 1.1 Yes hypothetical protein TT1380  2vzcA 2k2rA 127 129 2.6 Yes ALPHA-PARVIN  1jx6A 1zhhA 338 344 3.4 Yes LUXP PROTEIN  2guiA 2idoC 176 175 1.4 Yes DNA polymerase III epsilon subunit  1jf8A 1jfvA 130 129 2.0 Yes arsenate reductase  2qrlA 2qrjA 371 365 1.6 Yes Saccharopine dehydrogenase, NAD+, L-lysine-forming  2f01A 2g5lA 121 121 2.8 Yes Streptavidin  2iu5A 2iu5B 180 178 1.1 Yes HYPOTHETICAL PROTEIN YCEG  2hc1A 2ahsB 290 272 1.9 Yes Receptor-type tyrosine-protein phosphatase beta  2iyvA 2iyzA 179 168 4.1 Yes SHIKIMATE KINASE  1nnfA 1mrpA 308 309 2.5 Yes Iron-utilization periplasmic protein  2dhoA 2ickA 215 220 1.2 Yes Isopentenyl-diphosphate delta-isomerase 1  3d9aL 1rihL 213 212 3.1 No Light Chain of HyHel10 Antibody Fragment (Fab)  1ekqA 1esqA 253 258 1.6 Yes HYDROXYETHYLTHIAZOLE KINASE  1t1uA 1q6xA 597 600 1.4 Yes Choline O-acetyltransferase  1q35A 1si1A 317 316 2.5 Yes iron binding protein FbpA  1gvjA 1r36A 142 140 8.0 Yes C-ETS-1 PROTEIN  1shuX 1shtX 181 177 1.3 Yes Anthrax toxin receptor 2  1w5qA 1b4kB 321 320 1.4 Yes DELTA-AMINOLEVULINIC ACID DEHYDRATASE  2uytA 2cgkB 479 479 1.7 Yes RHAMNULOKINASE  1icmA 1t8vA 131 131 3.3 Yes INTESTINAL FATTY ACID BINDING PROTEIN  2pieA 2cswA 132 145 1.1 Yes E3 ubiquitin-protein ligase RNF8  2rdgA 2rdhB 192 190 1.6 Yes Superantigen-like protein 11  1qksA 1hcmA 559 526 6.5 Yes CYTOCHROME CD1 NITRITE REDUCTASE  2c1vA 2c1uA 335 329 5.7 Yes DI-HAEM CYTOCHROME C PEROXIDASE  1wbeA 2evlA 204 205 1.1 Yes GLYCOLIPID TRANSFER PROTEIN  3dwvA 2vupA 163 164 4.9 Yes Glutathione peroxidase-like protein  3d9nA 2diwA 138 152 1.0 Yes RNA-binding protein 16  1kq6A 1gd5A 136 130 3.3 Yes neutrophil cytosol factor 1  1v6sA 2ie8A 390 390 1.7 Yes Phosphoglycerate kinase  2grcA 2h60A 121 105 3.9 Yes Probable global transcription activator SNF2L4  2yveA 2zozB 175 181 1.7 Yes Transcriptional regulator  1k55A 2hp5B 244 238 1.6 Yes Beta lactamase OXA-10  1tbfA 1t9rA 326 301 2.8 Yes cGMP-specific 3',5'-cyclic phosphodiesterase  1iuqA 1k30A 350 363 1.0 Yes Glycerol-3-Phosphate Acyltransferase  1i0rA 1i0sB 161 168 1.8 Yes CONSERVED HYPOTHETICAL PROTEIN  1sfxA 1sfxB 105 102 1.6 Yes Conserved hypothetical protein AF2008  1o7qA 2vxmD 287 260 4.0 Yes N-ACETYLLACTOSAMINIDE ALPHA-1,3-GALACTOSYLTRANSFERASE  2yykA 2yymA 472 476 1.5 Yes 4-hydroxyphenylacetate-3-hydroxylase  1i52A 1vgtB 225 217 1.4 Yes 4-DIPHOSPHOCYTIDYL-2-C-METHYLERYTHRITOL SYNTHASE  1dd9A 1eqnB 310 304 1.1 Yes DNA PRIMASE  2avkA 2awcA 133 130 1.1 Yes hemerythrin-like domain protein DcrH  3b8zA 3b8zB 217 217 1.1 Yes protein ADAMTS-5  1z2uA 1w4uA 147 147 2.5 Yes Ubiquitin-conjugating enzyme E2 2  1jfbA 1xqdA 399 399 1.5 Yes nitric-oxide reductase cytochrome P450 55A1  2halA 1qa7B 212 218 2.7 No Hepatitis A Protease 3C  2fcjA 2i5rC 114 117 1.1 Yes small TOPRIM domain protein  1ie9A 2zmjA 255 240 1.2 Yes VITAMIN D3 RECEPTOR  1f86A 3cykD 115 115 1.5 Yes TRANSTHYRETIN THR119MET VARIANT  3covA 2a7xA 286 277 1.7 Yes Pantothenate synthetase  2ov0A 1mdaA 105 103 2.6 Yes Amicyanin  2oaaA 2oa9A 246 243 8.0 Yes R.MvaI  3duwA 3dulB 218 205 2.2 Yes O-methyltransferase, putative  1zhxA 1zi7A 434 402 2.9 Yes KES1 protein  2j0iA 2bvaA 289 272 2.4 Yes SERINE/THREONINE-PROTEIN KINASE PAK 4  2oocA 2oocB 103 104 1.7 Yes Histidine phosphotransferase  1wkqA 1tiyA 156 155 1.0 Yes Guanine deaminase  2hewF 2heyG 128 134 1.6 No Tumor necrosis factor ligand superfamily member 4  2cf7A 1umnG 156 165 1.5 Yes DPR  2oqzA 2oqwA 204 198 1.0 Yes Sortase B  2gkeA 2q9jA 274 273 3.0 Yes Diaminopimelate epimerase  1rg8A 1ry7A 137 151 3.8 Yes Heparin-binding growth factor 1  2fh1A 1p8xC 321 324 1.3 Yes Gelsolin  1idpA 1stdA 147 162 1.0 Yes SCYTALONE DEHYDRATASE  1f9yA 1eq0A 158 158 4.3 Yes 6-HYDROXYMETHYL-7,8-DIHYDROPTERIN PYROPHOSPHOKINASE  3ckcA 3ckcB 500 499 1.0 Yes SusD  3f43A 2ka5A 109 125 1.6 Yes Putative anti-sigma factor antagonist TM1081  1x91A 1x90B 149 147 10.6 Yes invertase/pectin methylesterase inhibitor family protein  1rhsA 1orbA 292 293 1.1 Yes SULFUR-SUBSTITUTED RHODANESE  2absA 1liiA 340 331 3.4 Yes adenosine kinase  1lkkA 1bhhA 105 104 1.3 Yes HUMAN P56 TYROSINE KINASE  1hd2A 1oc3C 161 161 8.6 Yes PEROXIREDOXIN 5 RESIDUES 54-214  2vlqB 1v13A 134 120 1.8 Yes COLICIN E9  2cibA 2vkuA 420 445 1.1 Yes CYTOCHROME P450 51  2o02A 1ib1A 224 227 1.7 Yes 14-3-3 protein zeta/delta  1z0wA 1z0gF 203 196 1.0 Yes Putative protease La homolog type  1f74A 1f5zD 293 293 2.0 Yes N-ACETYL-NEURAMINATE LYASE  1omrA 1ikuA 201 188 10.7 Yes recoverin  1jl1A 1rchA 152 155 2.3 Yes RIBONUCLEASE HI  3bmoA 2c7vC 255 258 1.1 Yes Pteridine reductase  1dj0A 2nreA 264 259 1.0 Yes PSEUDOURIDINE SYNTHASE I  1sz7A 2cfhB 159 161 1.1 Yes Trafficking protein particle complex subunit 3  2carA 2j4eD 194 194 2.1 Yes INOSINE TRIPHOSPHATE PYROPHOSPHATASE  1f46A 1f7xA 139 144 1.8 Yes CELL DIVISION PROTEIN ZIPA  2dxuA 2dz9B 231 235 1.3 Yes biotin--[acetyl-CoA-carboxylase] ligase  1ijyA 1ijyB 122 122 1.1 Yes FRIZZLED HOMOLOG 8  2zouA 2zouB 135 140 1.1 Yes Spondin-1  1tjyA 1tm2A 316 314 2.4 Yes sugar transport protein  1qgvA 1syxE 131 135 1.6 Yes SPLICEOSOMAL PROTEIN U5-15KD  1ntvA 1oqnB 152 151 3.0 Yes Disabled homolog 1  2qcfA 2jgyA 257 249 1.8 Yes Uridine 5'-monophosphate synthase (UMP synthase)  1p0zA 2v9aB 131 106 2.7 Yes Sensor kinase citA  2zcmA 2zcnD 173 184 1.9 Yes Biofilm operon icaABCD HTH-type negative transcriptional regulator icaR  1vhwA 1vhjD 237 232 1.0 Yes purine nucleoside phosphorylase  1m40A 1pzpA 263 263 1.0 Yes BETA-LACTAMASE TEM  1dqzA 1dqyA 280 282 2.5 Yes ANTIGEN 85-C  3f6yA 2o3sB 235 252 2.8 Yes ADP-ribosyl cyclase 1  1ifrA 1ivtA 113 122 2.2 Yes Lamin A/C  2imsA 2yv6A 159 155 1.2 Yes Apoptosis regulator BAK  1g6hA 1gajA 254 253 1.3 Yes HIGH-AFFINITY BRANCHED-CHAIN AMINO ACID TRANSPORT ATP-BINDING PROTEIN  2inwA 2inwB 115 114 3.3 Yes Putative structural protein  2nvhA 1hibA 152 150 1.4 Yes Interleukin-1 beta  1k3xA 1q39A 253 257 8.5 Yes Endonuclease VIII  2vn6A 2vn5C 151 148 2.1 Yes SCAFFOLDING PROTEIN  2fojA 2f1wA 137 144 2.7 Yes Ubiquitin carboxyl-terminal hydrolase 7  3c9aA 3cguA 208 207 7.4 No Protein giant-lens  1oi0A 1oi0B 108 106 2.2 Yes HYPOTHETICAL PROTEIN AF2198  2gzsA 2gzrA 245 238 1.1 Yes IroE protein  1u7gA 2nuuB 372 406 1.6 Yes Probable ammonium transporter  1nn5A 1e9fA 204 202 1.2 Yes Similar to deoxythymidylate kinase (thymidylate kinase)  2qimA 1xdfB 157 157 3.3 Yes PR10.2B  1nzjA 2zlzA 273 297 1.0 Yes Hypothetical protein yadB  2elcA 2elcC 329 329 1.5 Yes Anthranilate phosphoribosyltransferase  1i12A 1i21X 154 146 1.0 Yes GLUCOSAMINE-PHOSPHATE N-ACETYLTRANSFERASE  2bmwA 1go2A 295 295 1.0 Yes FERREDOXIN--NADP REDUCTASE  1n7oA 2brwA 721 722 2.2 Yes hyaluronidase  2cxyA 2eh9A 113 109 3.1 Yes BAF250b subunit  1so7A 2f25A 361 373 2.2 Yes Sialidase 2  1pqhA 1pyqB 115 116 2.1 Yes Aspartate 1-decarboxylase  2izrA 2chlA 298 298 1.1 Yes CASEIN KINASE I ISOFORM GAMMA-3  2jjuA 2d9cA 107 136 1.3 Yes SIGNAL REGULATORY PROTEIN BETA-1  2odiA 2odhA 232 233 3.1 Yes R.BcnI  1gciA 1ah2A 269 269 1.8 Yes SUBTILISIN  1po5A 2bdmA 465 465 5.6 Yes Cytochrome P450 2B4  2ob3A 1ptaA 329 318 3.3 Yes Parathion hydrolase  1n08A 1n05A 154 142 2.2 Yes putative riboflavin kinase  2gxgA 2yr2A 140 142 1.0 Yes 146aa long hypothetical transcriptional regulator  1yrcA 1k2oA 405 406 2.1 Yes Cytochrome P450-cam  2j9cA 2j9dE 115 114 4.8 Yes HYPOTHETICAL NITROGEN REGULATORY PII-LIKE PROTEIN MJ0059  1rdqE 1sykA 340 348 4.6 Yes cAMP-dependent protein kinase, alpha-catalytic subunit  1gp0A 2cnjD 133 144 1.2 Yes CATION-INDEPENDENT MANNOSE-6-PHOSPHATE RECEPTOR  1r8sA 1u81A 160 164 4.9 Yes ADP-ribosylation factor 1  1yc5A 2h2iA 234 244 1.6 Yes NAD-dependent deacetylase  2ce2X 1nvvR 166 166 3.8 Yes GTPASE HRAS  1h4xA 1h4yA 110 115 1.6 Yes ANTI-SIGMA F FACTOR ANTAGONIST  1pkhA 2hxdA 182 199 2.7 Yes Bifunctional deaminase/diphosphatase  2p7oA 2p7pD 127 125 4.5 Yes Glyoxalase family protein  2oxgB 2ox5D 110 110 1.6 Yes SoxY protein  1vdwA 1vdwB 248 248 1.8 Yes hypothetical protein PH1897  2tnfA 2tnfC 148 148 1.2 Yes TUMOR NECROSIS FACTOR ALPHA  2cnqA 1obgA 301 305 1.8 Yes PHOSPHORIBOSYLAMINOIMIDAZOLE-SUCCINOCARBOXAMIDE SYNTHASE  3dskA 2rchB 466 465 1.3 Yes Cytochrome P450 74A, chloroplast  2grrB 2io2C 157 156 17.3 No Ran GTPase-activating protein 1  2ci1A 2ci6A 273 274 1.1 Yes NG,NG-DIMETHYLARGININE DIMETHYLAMINOHYDROLASE 1  1j0pA 1it1A 107 107 1.2 Yes Cytochrome c3  2v6vA 2czoA 133 130 3.8 Yes BUD EMERGENCE PROTEIN 1  1r29A 3bimA 122 125 1.2 Yes B-cell lymphoma 6 protein  1m4iA 1m44B 181 180 1.8 Yes Aminoglycoside 2'-N-acetyltransferase  2q7wA 1tokA 378 395 1.7 Yes Aspartate aminotransferase  2uuqA 2uvnB 394 392 2.3 Yes CYTOCHROME P450 130  1f0lA 1xdtT 520 518 15.7 No DIPHTHERIA TOXIN  2uu8A 1enqB 237 233 1.3 Yes CONCANAVALIN  2ixmA 2hv6B 300 295 1.7 Yes SERINE/THREONINE-PROTEIN PHOSPHATASE 2A REGULATORY SUBUNIT B  1jr8A 1jraC 105 105 1.3 Yes Erv2 PROTEIN, mitochondrial  1n62B 1n5wE 804 795 1.5 Yes Carbon monoxide dehydrogenase large chain  2hbtA 2hbuA 224 219 1.1 Yes Egl nine homolog 1  2cjtA 2cjtC 118 127 3.9 No UNC-13 HOMOLOG A  1nwzA 3phyA 125 125 3.0 Yes Photoactive yellow protein  2h2zA 2qcyA 306 306 5.2 Yes Replicase polyprotein 1ab  1usgA 1usiA 346 347 7.1 Yes LEUCINE-SPECIFIC BINDING PROTEIN  2fcwA 2ftuA 106 118 4.5 Yes Alpha-2-macroglobulin receptor-associated protein  2g7bA 1blrA 137 137 3.5 Yes Cellular retinoic acid-binding protein 2  1tvgA 1xpwA 136 143 1.3 Yes LOC51668 protein  2nmlA 1wwqA 100 111 2.0 Yes Enhancer of rudimentary homolog  3bi1A 1z8lA 694 695 1.2 Yes Glutamate carboxypeptidase 2  1kfcA 1a5sA 254 258 1.4 Yes TRYPTOPHAN SYNTHASE ALPHA CHAIN  1xs0A 1gpqA 128 127 1.4 Yes Inhibitor of vertebrate lysozyme  2vclA 2vckB 199 203 1.2 Yes CYANOBACTERIAL PHYCOERYTHROBILIN  1e5kA 1fr9A 188 185 1.1 Yes MOLYBDOPTERIN-GUANINE DINUCLEOTIDE BIOSYNTHESIS PROTEIN A  1lo7A 1bvqA 140 139 1.3 Yes 4-hydroxybenzoyl-CoA Thioesterase  3bhyA 2j90B 268 262 6.6 Yes Death-associated protein kinase 3  1wdpA 1q6fA 493 490 1.0 Yes Beta-amylase  1wpuA 1wpsB 147 144 2.5 Yes Hut operon positive regulatory protein  1xg0C 1xg0D 173 176 1.8 Yes B-phycoerythrin beta chain  2gu9A 2ilbA 111 111 2.4 Yes tetracenomycin polyketide synthesis protein  1szhA 1szhB 147 143 1.2 No Her-1 protein  2w40A 2w41B 501 501 3.6 Yes GLYCEROL KINASE, PUTATIVE  1q7eA 1pt5A 401 415 1.1 Yes Hypothetical protein yfdW  2fr5A 1zabD 136 137 1.2 Yes Cytidine deaminase  1kafA 1kafE 108 103 1.1 No Transcription regulatory protein MOTA  1kqpA 1ih8B 271 247 1.3 Yes NH(3)-dependent NAD(+) synthetase  2je6B 2c37N 234 241 1.3 Yes EXOSOME COMPLEX EXONUCLEASE 1  1f7dA 1f7oA 118 116 1.3 Yes POL POLYPROTEIN  1m1fA 2c06B 107 110 1.7 Yes Kid toxin protein  1t4bA 1gl3B 367 367 1.3 Yes Aspartate-semialdehyde dehydrogenase  2a0bA 1fr0A 118 125 1.4 Yes HPT DOMAIN  1a2pA 1yvsA 108 108 17.2 Yes BARNASE  2jhfA 7adhA 374 374 1.4 Yes ALCOHOL DEHYDROGENASE E CHAIN  2b02A 1x0oA 108 119 2.1 Yes Aryl hydrocarbon receptor nuclear translocator  2f1kA 2f1kC 277 276 1.2 Yes prephenate dehydrogenase  1gwmA 1w90A 153 151 1.6 No NON-CATALYTIC PROTEIN 1  2oznA 2o4eA 133 165 1.4 No O-GlcNAcase nagJ  2ev1A 2ev4A 185 186 1.3 Yes Hypothetical protein Rv1264/MT1302  1uwkA 2v7gC 554 553 3.9 Yes UROCANATE HYDRATASE  2h8eA 1q8rA 120 118 1.1 Yes Crossover junction endodeoxyribonuclease rusA  3vubA 2vubH 101 101 1.8 Yes CCDB  2v03A 2bhtB 293 280 1.6 Yes CYSTEINE SYNTHASE B  1evlA 1evkA 401 401 1.6 Yes THREONYL-TRNA SYNTHETASE  2jhmF 2d39C 217 212 2.5 Yes FICOLIN-1  2axwA 2ixqA 134 142 3.5 No DraD invasin  2v9lA 2uyuA 274 274 1.0 Yes RHAMNULOSE-1-PHOSPHATE ALDOLASE  1lf7A 2qosC 164 173 1.3 Yes Complement Protein C8gamma  1ymtA 3f7dA 235 237 2.3 Yes Steroidogenic factor 1  2h3lA 1n7tA 103 103 5.0 Yes LAP2 protein  256bA 1yzaA 106 106 9.4 Yes CYTOCHROME B562  2o6pA 2o6pB 123 119 1.8 Yes Iron-regulated surface determinant protein C  1w1hA 1w1hB 147 151 4.1 Yes 3-PHOSPHOINOSITIDE DEPENDENT PROTEIN KINASE-1  2qp8A 2qu2A 390 364 1.5 Yes Beta-secretase 1  2i5vO 2ol8O 246 244 2.8 Yes Outer surface protein A  2it2A 2it3B 182 188 1.7 Yes UPF0130 protein PH1069  2cvdA 2vcqA 198 198 1.1 Yes Glutathione-requiring prostaglandin D synthase  2pfiA 2pfiB 145 148 1.1 Yes Chloride channel protein ClC-Ka  2ij2A 2uwhD 450 458 1.9 Yes Cytochrome P450 BM3  2g5rA 2hrlA 115 116 1.8 Yes Sialic acid-binding Ig-like lectin 7  1s2oA 1tj4A 244 244 3.3 Yes sucrose-phosphatase  2hzlA 2hzkC 334 325 1.8 Yes TRAP-T family sorbitol/mannitol transporter, periplasmic binding protein, SmoM  2ilkA 1lk3B 155 136 18.2 Yes INTERLEUKIN-10  1p6oA 1ysbB 156 161 1.3 Yes Cytosine deaminase  1zk4A 1zk3C 251 251 1.0 Yes R-specific alcohol dehydrogenase  1ypqA 1ypuB 131 135 1.2 Yes oxidised low density lipoprotein (lectin-like) receptor 1  1x8qA 2np1B 184 184 1.4 Yes Nitrophorin 4  2r4iA 2r4iC 120 119 1.3 Yes Uncharacterized protein  2orwA 2qpoA 171 153 2.6 Yes Thymidine kinase  1unqA 1unpA 117 119 1.9 Yes RAC-ALPHA SERINE/THREONINE KINASE  1mfmA 1rk7A 153 153 3.9 Yes COPPER,ZINC SUPEROXIDE DISMUTASE  2h6fA 1tnoK 315 314 1.1 Yes Protein farnesyltransferase/geranylgeranyltransferase type I alpha subunit  2pkfA 2pkkA 324 323 4.9 Yes Adenosine kinase  1hx0A 1dhkA 495 495 1.4 Yes ALPHA AMYLASE (PPA)  1f9zA 1fa5B 128 128 3.2 Yes GLYOXALASE I  1dcsA 1w2oA 279 276 1.8 Yes DEACETOXYCEPHALOSPORIN C SYNTHASE  1i1jA 1hjdA 106 101 2.9 Yes MELANOMA DERIVED GROWTH REGULATORY PROTEIN  1o08A 1lvhB 221 220 3.8 Yes beta-phosphoglucomutase  1gv9A 1r1zB 223 247 1.1 Yes P58/ERGIC-53  1m0kA 1brdA 222 177 2.1 Yes bacteriorhodopsin  1rkuA 1rkuB 205 205 1.2 Yes homoserine kinase  2c78A 1aipA 397 373 9.7 Yes ELONGATION FACTOR TU-A  1svsA 1y3aD 316 304 2.2 Yes Guanine nucleotide-binding protein G(i), alpha-1 subunit  2a35A 2a35B 208 208 1.1 Yes hypothetical protein PA4017  3dg9A 2vlbC 232 236 3.0 Yes Arylmalonate decarboxylase  2rl8A 2rl7A 148 148 3.0 Yes Cation-dependent mannose-6-phosphate receptor  1flmA 1axjA 122 122 2.1 Yes FMN-BINDING PROTEIN  1dy5A 1a2wB 123 124 15.0 Yes RIBONUCLEASE A  2vukA 2fejA 195 204 1.7 Yes CELLULAR TUMOR ANTIGEN P53  1hw1A 1h9gA 226 223 1.7 Yes FATTY ACID METABOLISM REGULATOR PROTEIN  1a62A 1a63A 122 130 1.8 Yes RHO  1thfD 1gpwC 253 253 3.8 Yes HISF PROTEIN  3d9xA 3d9xB 114 111 1.4 Yes Adhesin A  3f9mA 1v4tA 451 424 9.4 Yes Glucokinase  1n40A 2ij5E 395 387 1.4 Yes Cytochrome P450 121  1t3yA 1wm4A 131 142 2.5 Yes Coactosin-like protein  2z26A 2eg7B 343 342 1.5 Yes Dihydroorotase  1nycA 1qwxA 109 105 1.2 No cysteine protease inhibitor  3fcxA 3fcxB 268 275 1.7 Yes S-formylglutathione hydrolase  2arcA 1xjaD 161 165 2.9 Yes ARABINOSE OPERON REGULATORY PROTEIN  1pz7A 1q56A 188 195 3.0 Yes Agrin  2h8gA 2qsuA 246 247 1.8 Yes 5'-Methylthioadenosine Nucleosidase  3bpvA 3bpxB 137 138 1.9 Yes Transcriptional regulator  2qolA 2qoqA 273 284 1.4 Yes Ephrin receptor  2r3iA 1w98A 290 296 6.8 Yes Cell division protein kinase 2  1qveA 1hpwA 126 129 7.2 Yes Fimbrial protein  2qxfA 1fxxA 432 459 1.0 Yes Exodeoxyribonuclease I  1cxqA 1cxuA 143 143 1.6 Yes AVIAN SARCOMA VIRUS INTEGRASE  1gk9A 1e3aA 208 258 1.1 Yes PENICILLIN G ACYLASE ALPHA SUBUNIT  2gyqA 2gyqB 162 157 1.2 Yes ycfI, putative structural protein  3by8A 1ojgA 133 136 3.6 Yes Sensor protein dcuS  1jkxA 1garA 209 205 2.2 Yes PHOSPHORIBOSYLGLYCINAMIDE FORMYLTRANSFERASE  1x6zA 2py0A 120 120 1.1 Yes Fimbrial protein  2py5A 2pyjB 564 572 2.8 Yes DNA polymerase  1xjuA 1xjuB 156 155 2.6 Yes Lysozyme  1oxxK 1oxuC 352 353 1.5 Yes ABC transporter, ATP binding protein  1zzwA 1zzwB 147 147 1.0 Yes Dual specificity protein phosphatase 10  1xk7A 1xvvA 388 402 1.6 Yes Crotonobetainyl-CoA:carnitine CoA-transferase  3e2oA 1dseA 290 292 1.3 Yes Cytochrome c peroxidase  1ra0A 1r9zA 423 423 1.1 Yes Cytosine deaminase  1mf7A 1n9zA 194 184 3.0 Yes INTEGRIN ALPHA M  2iz6A 2iz5D 159 160 1.1 Yes MOLYBDENUM COFACTOR CARRIER PROTEIN  1j1nA 1kwhA 492 492 3.8 Yes AlgQ2  2qf4A 2qf5A 170 158 1.1 Yes Cell shape determining protein MreC  2p0sA 2p0sB 128 120 2.6 No ABC transporter, permease protein, putative  1pvmA 2qh1B 178 178 1.0 Yes conserved hypothetical protein Ta0289  2vb1A 1lkrB 129 129 1.7 Yes LYSOZYME C  2g2uB 2g2wB 165 164 1.9 No Beta-lactamase inhibitory protein  1nuyA 1eyjB 328 327 5.0 Yes Fructose-1,6-bisphosphatase  1ek6A 1ek5A 346 346 1.4 Yes UDP-GALACTOSE 4-EPIMERASE  1s1fA 1se6B 397 402 2.1 Yes putative cytochrome P450  2au7A 1mjyB 175 175 1.2 Yes Inorganic pyrophosphatase  1tt8A 2ahcB 164 164 1.3 Yes Chorismate-pyruvate lyase  1ryoA 1bp5B 324 328 6.8 Yes Serotransferrin  2fj8A 1tx6J 120 114 1.5 Yes Bowman-Birk type trypsin inhibitor  1qv1A 1sl7A 187 173 3.0 Yes Obelin  1xd3A 1uchA 229 206 1.7 Yes Ubiquitin Carboxyl-terminal esterase L3  2o0bA 2bjbA 424 422 4.1 Yes 3-phosphoshikimate 1-carboxyvinyltransferase  1jhgA 1mi7R 101 103 20.9 Yes TRP OPERON REPRESSOR  1vhtA 1viyC 207 207 2.6 Yes Dephospho-CoA kinase  2lisA 2lynA 131 131 2.8 Yes SPERM LYSIN  2pqcA 2gg4A 445 443 4.1 Yes 3-phosphoshikimate 1-carboxyvinyltransferase  1uylA 3ekoB 208 217 1.7 Yes HEAT SHOCK PROTEIN HSP 90-ALPHA  2nliA 2j6xC 352 368 2.0 Yes Lactate oxidase  2hhvA 1lv5B 580 580 2.2 Yes DNA Polymerase I  1y93A 2k9cA 158 152 3.7 Yes Macrophage metalloelastase  1o8xA 1okdA 144 148 1.7 Yes TRYPAREDOXIN  2z6oA 2k07A 166 175 2.2 Yes Ufm1-conjugating enzyme 1  1irdA 1z8uD 141 135 3.3 Yes Hemoglobin alpha chain  2b3gA 1ewiA 117 114 6.5 Yes Replication protein A 70 kDa DNA-binding subunit  1iomA 1ixeB 374 370 2.9 Yes CITRATE SYNTHASE  1p5zB 2zi6C 229 216 1.8 Yes Deoxycytidine kinase  1m1nA 1l5hA 477 405 2.6 Yes Nitrogenase molybdenum-iron protein alpha chain  2v8iA 2v8jA 543 535 1.8 Yes PECTATE LYASE  1t2dA 2a92C 315 317 1.8 Yes L-lactate dehydrogenase  2e2rA 1s9qB 228 216 6.0 Yes Estrogen-related receptor gamma  2a4xA 1kmzA 131 125 12.8 Yes Mitomycin-Binding Protein  1ixhA 1oibB 321 321 3.1 Yes PHOSPHATE-BINDING PROTEIN  1nnlA 1l8lB 205 222 1.2 Yes L-3-phosphoserine phosphatase  1q1fA 1oj6D 148 150 1.7 Yes Neuroglobin  1gu2A 1e8eA 124 124 1.6 Yes CYTOCHROME C'  1ynpA 1ynqB 282 297 1.8 Yes oxidoreductase  2p45B 2p49B 116 121 1.1 Yes ANTIBODY CAB-RN05  3bhdA 3bhdB 208 204 2.3 Yes Thiamine triphosphatase  1kmtA 1gdfA 138 145 1.4 Yes Rho GDP-dissociation inhibitor 1  2hxmA 1q3fA 223 223 1.2 Yes Uracil-DNA glycosylase  1v8cA 1v8cC 165 165 1.1 Yes MoaD related protein  1wcwA 3d8rA 254 252 8.9 Yes Uroporphyrinogen III synthase  1wddA 1ej7L 464 457 3.6 Yes Ribulose bisphosphate carboxylase large chain  1l3kA 1po6A 163 183 2.1 Yes HETEROGENEOUS NUCLEAR RIBONUCLEOPROTEIN A1  2f23A 2etnA 154 154 2.1 Yes Anti-cleavage anti-greA transcription factor gfh1  1o6vA 2omwA 464 461 4.1 Yes INTERNALIN A  1dfmA 1es8A 218 193 4.8 No ENDONUCLEASE BGLII  1n3lA 1q11A 332 329 1.4 Yes tyrosyl-tRNA synthetase  2bw4A 1niaA 334 333 2.1 Yes COPPER-CONTAINING NITRITE REDUCTASE  1tjxA 1k5wA 157 148 1.0 Yes similar to synaptotagminI/p65  1ey4A 1sndA 136 129 10.1 Yes STAPHYLOCOCCAL NUCLEASE  2d1eA 2dkeA 243 241 1.2 Yes Phycocyanobilin:ferredoxin oxidoreductase  2eplX 2epnA 614 623 1.0 Yes N-acetyl-beta-D-glucosaminidase  1fm0E 1nvjD 142 135 1.9 Yes MOLYBDOPTERIN CONVERTIN FACTOR, SUBUNIT 2  1ccwA 1b1aA 137 137 5.2 Yes GLUTAMATE MUTASE  1whiA 1c04D 122 122 1.1 Yes RIBOSOMAL PROTEIN L14  2r9fA 1kxrA 322 320 1.2 Yes Calpain-1 catalytic subunit  1ejdA 1uaeA 418 418 2.3 Yes UDP-N-ACETYLGLUCOSAMINE ENOLPYRUVYLTRANSFERASE  2nszA 2hm8A 129 136 1.8 Yes Programmed cell death protein 4  1wmwA 1wmwD 328 324 2.5 Yes geranylgeranyl diphosphate synthetase  1vlyA 1nrkA 314 315 1.3 Yes Unknown protein from 2D-page  1my7A 1my5B 107 101 1.3 Yes NF-kappaB p65 (RelA) subunit  2nw2B 2nx5E 243 243 1.0 Yes ELS4 TCR beta chain  1f2tB 1f2uB 143 145 3.2 Yes RAD50 ABC-ATPASE  2b4hA 2b4iC 227 232 6.8 Yes Outer capsid protein VP4  2z0jA 2z0jG 237 237 1.1 Yes Putative uncharacterized protein TTHA1438  1xubA 1u1wB 278 278 1.3 Yes Phenazine biosynthesis protein phzF  1m9zA 1ploA 105 122 1.6 Yes TGF-BETA RECEPTOR TYPE II  1jf3A 1vrfA 147 147 1.6 Yes monomer hemoglobin component III  2ozjA 2ozjB 106 106 1.1 Yes Cupin 2, conserved barrel  1t6cA 1t6dB 306 295 1.6 Yes exopolyphosphatase  2fulA 2fulE 161 164 2.0 Yes Eukaryotic translation initiation factor 5  3c8cA 3c8cB 239 239 1.1 Yes Methyl-accepting chemotaxis protein  1xlqA 1gpxA 106 106 3.0 Yes Putidaredoxin  3eqxA 3eqxB 362 358 1.5 Yes FIC DOMAIN CONTAINING TRANSCRIPTIONAL REGULATOR  2zs0D 2zfoD 145 145 1.3 Yes Extracellular giant hemoglobin major globin subunit B1  1t7rA 1i38A 250 247 1.1 Yes Androgen receptor  2ppnA 1fkrA 107 107 1.4 Yes FK506-binding protein 1A  2jg2A 2jgtB 398 363 1.7 Yes SERINE PALMITOYLTRANSFERASE  2oznB 2jnkA 131 140 2.8 No Hyalurononglucosaminidase  1i0vA 1iyyA 104 104 1.9 Yes GUANYL-SPECIFIC RIBONUCLEASE T1  1d4tA 1d1zC 104 101 1.2 Yes T CELL SIGNAL TRANSDUCTION MOLECULE SAP  2r5oA 2r5oB 167 166 1.8 Yes Putative ATP binding component of ABC-transporter  2nw0A 2nw0B 189 189 1.2 Yes PlyB  3b5mA 3b5mB 193 194 1.1 Yes Uncharacterized protein  1xgkA 2vuuB 325 319 1.0 Yes NITROGEN METABOLITE REPRESSION REGULATOR NMRA  3ci6A 3ci6B 151 159 1.6 Yes Phosphoenolpyruvate-protein phosphotransferase  1p5dX 1k2yX 454 459 2.0 Yes Phosphomannomutase  1i4uA 1gkaA 181 180 1.7 Yes CRUSTACYANIN  1ls1A 1rj9B 289 282 2.8 Yes SIGNAL RECOGNITION PARTICLE PROTEIN  2zkdA 2zkgD 210 184 1.2 Yes E3 ubiquitin-protein ligase UHRF1  2bwqA 1v27A 122 141 1.4 Yes REGULATING SYNAPTIC MEMBRANE EXOCYTOSIS PROTEIN 2  2z6rA 2huvB 264 265 1.2 Yes diphthine synthase  2pwoA 1afvA 144 151 4.0 No Gag-Pol polyprotein (Pr160Gag-Pol)  3buxB 1b47A 305 304 1.9 Yes E3 ubiquitin-protein ligase CBL  2fvyA 2qw1A 305 305 3.9 Yes D-galactose-binding periplasmic protein  1pb7A 1pbqB 281 266 2.5 Yes N-methyl-D-aspartate Receptor Subunit 1  2gj4A 1gpaA 803 828 3.2 Yes Glycogen phosphorylase, muscle form  1t9iA 2o7mB 153 153 4.0 Yes DNA endonuclease I-CreI  2fctA 2fcuA 304 309 1.2 Yes syringomycin biosynthesis enzyme 2  2akzA 1te6B 435 433 1.3 Yes Gamma enolase  1us0A 1xgdA 313 315 2.3 Yes ALDOSE REDUCTASE  1ew4A 1soyA 106 106 1.6 Yes CYAY PROTEIN  1a8dA 1fv3B 452 451 1.4 Yes TETANUS NEUROTOXIN  1odmA 1ipsA 329 328 1.4 Yes ISOPENICILLIN N SYNTHASE  3pviA 1ni0B 156 157 3.4 No PVUII ENDONUCLEASE  2zdpA 1sqeB 108 106 4.0 Yes Heme-degrading monooxygenase isdI  2fomB 1befA 150 177 9.3 Yes polyprotein  1xu9A 2belB 269 251 3.0 Yes Corticosteroid 11-beta-dehydrogenase, isozyme 1  1wvhA 2gjyA 132 144 1.5 Yes Tensin  2cuaA 2cuaB 122 132 1.5 Yes CUA  2pq8A 2givA 260 257 1.1 Yes Probable histone acetyltransferase MYST1  2oktA 2olaA 341 339 1.8 Yes O-succinylbenzoic acid synthetase  1zi8A 1zj4A 233 232 1.1 Yes Carboxymethylenebutenolidase  1je0A 1jdvE 226 227 1.5 Yes 5'-METHYLTHIOADENOSINE PHOSPHORYLASE  1r5lA 1oizA 247 266 1.8 Yes Alpha-tocopherol transfer protein  1yfqA 2i3sA 342 334 2.4 Yes Cell cycle arrest protein BUB3  1btkA 1bwnB 160 161 1.8 Yes BRUTON'S TYROSINE KINASE  1b8oA 1lvuB 280 277 1.9 Yes PURINE NUCLEOSIDE PHOSPHORYLASE  1w0pA 1kitA 753 757 1.0 No SIALIDASE  2fufA 2tbdA 124 134 1.7 Yes Large T antigen  1pkoA 3cspA 123 121 1.1 Yes Myelin Oligodendrocyte Glycoprotein  1zl0A 2aunA 306 294 1.1 Yes hypothetical protein PA5198  2b82A 1rmtA 211 210 2.1 Yes class B acid phosphatase  1n62A 1zxiD 161 158 1.3 Yes Carbon monoxide dehydrogenase small chain  1kyfA 2vj0A 247 246 1.2 Yes ALPHA-ADAPTIN C  1xg4A 1oqfA 287 290 2.5 Yes Probable methylisocitrate lyase  2gs5A 2hrxA 185 183 1.7 Yes Conserved hypothetical protein  1r0mA 2gggC 360 370 1.1 Yes N-acylamino acid racemase  1g3pA 3dgsA 191 186 2.0 No MINOR COAT PROTEIN  2olnA 2oloA 385 393 1.8 Yes nikD protein  2pn6A 2yx7A 151 151 4.5 Yes 150aa long hypothetical transcriptional regulator  3sebA 1sbbB 238 239 2.4 Yes STAPHYLOCOCCAL ENTEROTOXIN B  1sh8A 1sh8B 152 147 1.4 Yes hypothetical protein PA5026  2qfaA 2rawA 137 137 1.6 Yes Baculoviral IAP repeat-containing protein 5  1k7kA 2q16A 195 195 2.3 Yes Hypothetical protein yggV  2fxuA 3byhA 360 374 3.4 No Actin, alpha skeletal muscle  1sauA 2a5wB 114 114 1.7 Yes sulfite reductase, desulfoviridin-type subunit gamma  2z1eA 2z1fA 298 304 1.9 Yes Hydrogenase expression/formation protein HypE  1o97C 1o96A 251 261 1.1 Yes ELECTRON TRANSFERRING FLAVOPROTEIN BETA-SUBUNIT  1jatB 2gmiB 132 135 1.1 Yes Ubiquitin-Conjugating Enzyme Variant Mms2  1e7lA 1en7A 157 157 2.7 Yes RECOMBINATION ENDONUCLEASE VII  1gtvA 1w2hA 208 191 1.5 Yes THYMIDYLATE KINASE  2g3rA 1xniA 119 118 1.2 No Tumor suppressor p53-binding protein 1  2nw8A 3e08C 268 282 3.1 Yes Tryptophan 2,3-dioxygenase  1jetA 1rkmA 517 517 3.2 Yes OLIGO-PEPTIDE BINDING PROTEIN  3d32A 1klvA 118 100 2.7 Yes Gamma-aminobutyric acid receptor-associated protein  1pp0A 1vgfA 191 194 1.1 No volvatoxin A2  1n57A 1pv2G 272 260 1.1 Yes Chaperone Hsp31  1u4gA 1ezmA 298 298 1.2 Yes Elastase  1fsgA 1qk3B 230 228 6.4 Yes HYPOXANTHINE-GUANINE PHOSPHORIBOSYLTRANSFERASE  2z08A 2z3vA 123 137 2.0 Yes Universal stress protein family  2erfA 1z78A 209 206 1.3 Yes Thrombospondin-1  1nxmA 2ixlA 194 196 1.6 Yes dTDP-6-deoxy-D-xylo-4-hexulose 3,5-epimerase  1n8vA 1k19A 101 112 4.0 Yes chemosensory protein  2c61A 2rkwA 432 422 1.9 Yes A-TYPE ATP SYNTHASE NON-CATALYTIC SUBUNIT B |

**Table S3**. Dynamically important residues in myosin predicted by three perturbation-based scores and DynDom.

| score | residue numbers |
| --- | --- |
| $\delta f_{i}^{obs}$ | 35-37,47,80-83,85-88,90-96,105,108-109,112,114,116-120,122-125,155,176,178-179,181-186,188,226-227,233,236-239,256,457,465,468,470-472,474,476,478-481,483-499,651-652,654-658,672,676,679,681,684-685,688-689,692-696,738-746, |
| $\delta f_{i}$ | 34-37,49-52,79-82,85-87,89-91,93-96,105,108,116,118-120,123,152,154,173-179,181-183,185,233,237,239,241,263,265,395-397,451,453,457-459,468,471-484,486-491,493-494,497,499,510,514,551,572,588,623,625-626,648,652,654-656,672,676,680-681,684-685,692,695,738-745, |
|  | 35-37,47,80-83,85-91,93-96,102,105-109,111-112,114,116-120,122-123,125,152-156,174-179,181-183,185-186,188,233,453,457-459,468,470-476,478,480,482-489,491,493-494,497-499,572-574,652-658,669-670,672-674,676,678,680-681,683-685,695,739-740,743-745, |
| DynDom | 485,486,487,501,502,503,504,505,506,507,508,509,510,511,512,681,682,683,684,685,686,687,688,689,690,691,200,201,202,203,204,205,206,207,208,209,210,211,245,246,247,254,255,444,445,446,447,450,451,648,649, |

**Table S4**. Dynamically important residues in kinesin predicted by three perturbation-based scores.

| score | residue numbers |
| --- | --- |
| $\delta f_{i}^{obs}$ | 8-10,31-34,54-55,57,92,94-96,98,103,107,150,153,157,208,213,243,245,247,251-254,272,274,276-278,280,284,312,315,318-319,323,327-329,332,337,339,341,344,351, |
| $\delta f_{i}$ | 8-10,12,30-34,52,54-58,85,88-93,145-146,178-181,220-222,224-227,241-243,245-247,278,280,318-319,323,327,335,337,341, |
|  | 6,85,89-92,94,96,98,107-108,119-124,146-147,150,156-159,189,199,201-202,217,243,245,250,252-253,275,277-279,281-283,285-286,306-309,312,316,318, |
